# Ti Group Metallocene-Catalyzed Synthesis of 1-Hexene Dimers and Tetramers

**DOI:** 10.3390/molecules26092775

**Published:** 2021-05-08

**Authors:** Pavel V. Kovyazin, Almira Kh. Bikmeeva, Denis N. Islamov, Vasiliy M. Yanybin, Tatyana V. Tyumkina, Lyudmila V. Parfenova

**Affiliations:** Institute of Petrochemistry and Catalysis of Russian Academy of Sciences, Prospekt Oktyabrya, 141, 450075 Ufa, Russia; kpv38@mail.ru (P.V.K.); almira.bikmeeva@gmail.com (A.K.B.); islamov19@gmail.com (D.N.I.); vyanybin@gmail.com (V.M.Y.); ttvnmr@gmail.com (T.V.T.)

**Keywords:** metallocenes, metal hydrides, dimerization, nuclear magnetic resonance, density functional theory

## Abstract

1-Hexene transformations in the catalytic systems L_2_MCl_2_–XAlBu^i^_2_ (L = Cp, M = Ti, Zr, Hf; L = Ind, rac-H_4_C_2_[THInd]_2_, M = Zr; X = H, Bu ^i^) and [Cp_2_ZrH_2_]_2_-ClAlR_2_ activated by MMAO-12, B(C_6_F_5_)_3_, or (Ph_3_C)[B(C_6_F_5_)_4_] in chlorinated solvents (CH_2_Cl_2_, CHCl_3_, o-Cl_2_C_6_H_4_, ClCH_2_CH_2_Cl) were studied. The systems [Cp_2_ZrH_2_]_2_-MMAO-12, [Cp_2_ZrH_2_]_2_-ClAlBu^i^_2_-MMAO-12, or Cp_2_ZrCl_2_-HAlBu^i^_2_-MMAO-12 (B(C_6_F_5_)_3_) in CH_2_Cl_2_ showed the highest activity and selectivity towards the formation of vinylidene head-to-tail alkene dimers. The use of chloroform as a solvent provides further in situ dimer dimerization to give a tetramer yield of up to 89%. A study of the reaction of [Cp_2_ZrH_2_]_2_ or Cp_2_ZrCl_2_ with organoaluminum compounds and MMAO-12 by NMR spectroscopy confirmed the formation of Zr,Zr-hydride clusters as key intermediates of the alkene dimerization. The probable structure of the Zr,Zr-hydride clusters and ways of their generation in the catalytic systems were analyzed using a quantum chemical approach (DFT).

## 1. Introduction

Alkene dimers and oligomers represent a large class of compounds that are used as comonomers in ethylene polymerization and as raw materials for the production of adhesives, surfactants, fragrances, synthetic lubricating fuel additives, etc. [1,2,3,4,5,6]. The following industrial processes have been successfully developed and implemented for the production of olefin oligomers: (i) oligomerization of ethylene in the presence of triethylaluminum with subsequent oxidation to higher alcohols (Ziegler–Alfol process), (ii) Philips process of ethylene oligo—and polymerization on chromium catalysts, (iii) Ni-catalyzed synthesis of linear α-olefins via ethylene oligomerization (shell higher olefin process (SHOP)), (iv) oligomerization of ethylene to linear C4–C10 olefins by α-select technology (Axens) or α-SABLIN technology (Sabic and Linde) [7]. Currently, some research groups are developing strategies for the production of highly efficient jet and diesel fuels via oligomerization of alkenes (1-butene, 1-hexene) synthesized from renewable plant raw materials [2,8].

**Scheme 1:**
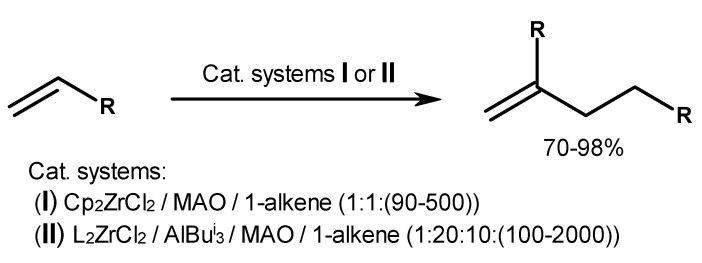
Zirconocene-catalyzed dimerization of terminal alkenes.

Among the catalysts, Ti group metallocene complexes showed high efficiency in alkene di-, oligo-, and polymerization [9,10,11,12]. In this way, the process of zirconocene-catalyzed dimerization of terminal alkenes to give products with a vinylidene moiety >C=CH_2_ is well-known (Scheme 1) [5,6,13,14,15,16,17,18,19,20].

Despite significant progress in this area, the mechanism of the dimerization reaction is not fully understood. Earlier, the hypothesis on the key role of the hydride cation [Cp_2_ZrH]^+^ was put forward [14]. Subsequently, it was postulated that bimetallic hydride complexes L_2_Zr(*µ*-H)_3_AlR_2_ can act as catalytically active species in the alkene dimerization [21,22]. Recently, we studied the systems Cp_2_ZrCl_2_-XAlBu^i^_2_ (X = H, Bu^i^) and [Cp_2_ZrH_2_]_2_-ClAlR_2_ (R = Me, Et, Bu^i^) activated by MMAO-12 (modified methylaluminoxane) or organoboron compounds and found new biszirconium hydride intermediates (Cp_2_ZrH_2_^.^Cp_2_ZrHCl^.^ClAlR_2_), which provide selective formation of head-to-tail dimerization products [19,20,23]. However, the questions on the possible structure of the key intermediates and their formation in the catalytic systems remain open.

Thus, our work was aimed at investigating the effect of transition metal *η*^5^-complexes and solvents (chlorine-containing solvents) on the activity, chemo- and regioselectivity of the systems L_2_MCl_2_–XAlBu^i^_2_ (L = Cp, M = Ti, Zr, Hf; L = Ind, rac-H_4_C_2_[THInd]_2_, M = Zr; X = H, Bu^i^) and [Cp_2_ZrH_2_]_2_-ClAlR_2_ activated by MMAO-12, B(C_6_F_5_)_3_, or (Ph_3_C)[B(C_6_F_5_)_4_] in alkene oligomerization. NMR studies and quantum chemical calculations were applied to establish the possible structure of the hydride intermediates formed in these systems. The results showed that the M,M-type bimetallic hydrides and their action mechanisms are unique for the dimerization reaction.

## 2. Results and Discussion

### 2.1. Transformations of 1-Hexene with Cp_2_MY_2_ (M = Ti, Zr, Hf; Y = H, Cl)-XAlBu^i^_2_ (X = H, Bu^i^) Catalytic Systems Activated by MMAO-12, B(C_6_F_5_)_3_, or (Ph_3_C)[B(C_6_F_5_)_4_]

**Scheme 2:**
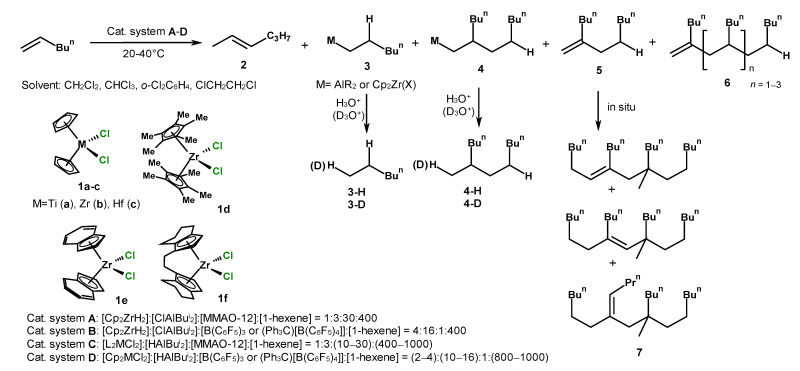
1-Hexene dimerization and oligomerization in catalytic systems **A**–**D**.

The conditions for the selective synthesis of vinylidene dimers in the presence of catalytic systems Cp_2_ZrY_2_ (Y = H, Cl)-XAlBu^i^_2_ (X = H, Bu^i^)-activator (Scheme 2, catalytic systems **A** and **B**), developed in our previous study, were taken as the starting point for the experiment (Table 1, entry 1) [19,20].

The reaction proceeds in toluene or benzene at 20–60 °C to give the target products in 81–98% yields within 5 to 150 min, depending on the type of activator (Table 1, entries 1, 15, 21, 27, and 43). The use of chlorine-containing solvents in these systems was found to accelerate the reaction. For example, the system [Cp_2_ZrH_2_]_2_-ClAlBu^i^_2_-MMAO-12 with a molar ratio [Zr]:[Al]:[MMAO‑12]:[1-hexene] = 1:3:30:400 at a temperature of 40 °C in CH_2_Cl_2_ gives 1-hexene dimer in a yield of more than 98% by the 15th minute of the reaction (Table 1, entry 2). When the reaction is carried out in chloroform, the relative content of dimers decreases to 92% (entry 4). Under the same conditions, the dimers produced in the first minutes of the reaction serve as substrates for the subsequent dimerization to afford dimers of dimer **7** in 75–79% yields in 180 min (entries 7, 8, Figures S26 and S27). According to ^1^H and ^13^C NMR data, the structures of these products correspond to those proposed earlier [16,22,24,25,26]. Dimerization of **5** occurs probably via participation of cationic species formed upon the reaction of CHCl_3_ with ClAlR_2_ or MMAO-12.

The replacement of MMAO-12 by B(C_6_F_5_)_3_ in the system [Cp_2_ZrH_2_]_2_-ClAlBu^i^_2_-activator leads to a significant reduction in substrate conversion. The reaction occurs only in chloroform, and the conversion of 1-hexene in 16 h is 75% with a 71% content of dimers in the product mixture (entries 18, 19). Unlike B(C_6_F_5_)_3_, compound (Ph_3_C)[B(C_6_F_5_)_4_] can activate the system [Cp_2_ZrH_2_]_2_-ClAlBu^i^_2_. Under these conditions, the conversion of 1-hexene in 3 h was higher in CH_2_Cl_2_ (99%, entry 26), and the reaction predominantly gave tetramer **7** in 69% yield. In contrast, in chloroform, the 1-hexene conversion was 81% in 16 h, and oligomeric products **6**, including compound **7**, prevailed in the product mixture (entries 24, 25).

The use of chlorine-containing solvents CH_2_Cl_2_ and CHCl_3_ in the case of the system [Cp_2_ZrH_2_]_2_-MMAO-12 at the ratio [Zr]:[MMAO-12]:[1-hexene] = 1:30:400 allows the reaction to be carried out in the absence of ClAlR_2_ with a >99% conversion of 1-hexene and up to 89–91% yield of dimer **5** (entries 9, 10). In *o*-dichlorobenzene and 1,2-dichloroethane, the reaction proceeds to a lower substrate conversion and a lower yield of the dimeric product (entries 11–14). The composition of the reaction products formed in the system [Cp_2_ZrH_2_]_2_-MMAO-12-1-hexene for 16 h in any of the solvents (CH_2_Cl_2_, CHCl_3_, *o*-Cl_2_C_6_H_4_, ClCH_2_CH_2_Cl) did not change significantly. Attempts to activate [Cp_2_ZrH_2_]_2_ with boron-containing activators in the absence of ClAlR_2_ were unsuccessful (entries 16, 17, 22, 23).

Thus, vinylidene dimer **5** is formed in a high yield and with high selectivity under the action of [Cp_2_ZrH_2_]_2_-ClAlBu^i^_2_-MMAO-12 or [Cp_2_ZrH_2_]_2_-MMAO-12 in CH_2_Cl_2_. Chloroform facilitates the formation of a non-classical tetramer of 1-hexene (**7**), which could be used as a component of lubricants [16,24,25,26].

The catalytic system based on Cp_2_ZrCl_2_, HAlBu^i^_2,_ and MMAO-12 also provides vinylidene dimers **5** in chlorinated solvents. In CH_2_Cl_2_ and CHCl_3_, the reaction proceeds in 30 min with the conversion of 1-hexene above 99% and the yield of dimers of 98% (entries 28, 34). Increasing 1-hexene initial concentration to 1000 equivalents leads to decreased alkene conversion to 82% and the yield of dimeric product to 80% in CH_2_Cl_2_. Under these conditions, the conversion and the yield of dimers are higher in CHCl_3_ than in CH_2_Cl_2_ and amount to >99% and 90–98% in 30 min, respectively (entries 32, 35).

The reaction carried out in the presence of Cp_2_ZrCl_2_ and MMAO-12 at the ratio [Zr]:[MMAO-12]:[1-hexene] = 1:30:400 in chlorinated solvents (CH_2_Cl_2_, *o*-Cl_2_C_6_H_4_, ClCH_2_CH_2_Cl) at 40 °C results in 91–96% yield of dimers (entries 30, 31, 39–42). Carrying out the reaction in chloroform gives dimers of dimer **7** in a yield of up to 89% (entry 37). A decrease in the relative amount of MMAO-12 in the system to [Zr]:[MMAO-12]:[1-hexene] = 1:10:400 provides the selective formation of dimerization products at the level of 91% within 16 h.

The highest yield of dimer **5** at the level of 99% is achieved by using a neutral boron-containing activator B(C_6_F_5_)_3_ in the system Cp_2_ZrCl_2_-HAlBu^i^_2_ at the ratio [Zr]:[Al]:[B(C_6_F_5_)_3_]:[1-hexene] = 4:16:1:1000 and in dichloromethane as a solvent (entry 44).

Activation of system Cp_2_ZrCl_2_-HAlBu^i^_2_ by (Ph_3_C)[B(C_6_F_5_)_4_] at a ratio [Zr]:[Al]:[(Ph_3_C)[B(C_6_F_5_)_4_] ]:[1-hexene] = 4:16:1:1000 and a temperature 40 °C affords alkene oligomers in both CH_2_Cl_2_ and CHCl_3_ (entries 50–52, 54, 55). Selective formation of a dimer (92%, entry 49) occurs in CH_2_Cl_2_ at 20 °C, with the reaction time being up to 180 min.

Subsequently, the content of dimers decreases to 55%, and the amount of tetramer **7** increases to 33% (entry 50). Using chloroform as a solvent raises the yield of **7** to 65–72% (entries 54, 55).

The activity of Cp_2_TiCl_2_ (**1a**) and Cp_2_HfCl_2_ (**1c**) in the studied systems was lower than that of Cp_2_ZrCl_2_ (**1b**). Indeed, the conversion of 1-hexene in CH_2_Cl_2_ at 40 °C was 80% in 60 min of the reaction in the case of the Ti complex (Table 2, entries 1) and 84% in 120 min for the Hf complex (entries 5). The use of chloroform as a solvent increases substrate conversion to 93% for Cp_2_TiCl_2_ and decreases the conversion to 60% for Cp_2_HfCl_2_ (entries 3,8). Moreover, these catalysts are significantly inferior in chemo- and regioselectivity to zirconocene dichloride. Thus, in the presence of Cp_2_TiCl_2_, the yield of oligomeric products **6** and tetramer **7**, which represent a mixture of regioisomers, increases in total to 89%. The use of Cp_2_HfCl_2_ in CH_2_Cl_2_ leads to increased the content of vinylidene oligomers up to 28–37%. In chloroform, the yield of regioisomeric oligomers also increases in the first 60 min of the reaction (entry 8). Further, the appearance of products of toluene mono-, di-, and trialkylation with 1-hexene was found (entries 9,10). It should be noted that in the presence of HAlBu^i^_2_, upon replacing MMAO-12 with boron-containing activators, the systems based on Cp_2_TiCl_2_ and Cp_2_HfCl_2_ completely lost their activity (Table 2, entries 11–14).

The effect of the ligand structure of zirconocenes on the activity of catalytic systems and the reaction route was also studied. For this purpose, we tested Zr η^5^-complexes (C_5_Me_5_)_2_ZrCl_2_ (**1d**), Ind_2_ZrCl_2_ (**1e**), and *rac*-H_4_C_2_(THInd)_2_ZrCl_2_ (**1f**) (Scheme 2) as components of the systems L_2_ZrCl_2_-HAlBu^i^_2_-MMAO-12 at molar ratio [Zr]:[Al]:[MMAO-12]:[1-hexene] = 1:3:30:400 and temperature of 40 °C. It turned out that (C_5_Me_5_)_2_ZrCl_2_ does not lead to the formation of the target products either in CH_2_Cl_2_ or in CHCl_3_ (Table 3, entries 1,2). In this case, 1-hexene was consumed for toluene alkylation (toluene is present in the system as a solvent for MMAO-12). The complex Ind_2_ZrCl_2_ showed lower activity than Cp_2_ZrCl_2_ (Table 3, entries 3–7) and shifted the reaction route toward the oligomerization (up to 87%) both in CH_2_Cl_2_ and CHCl_3_. *Ansa*-zirconocene *rac*-H_4_C_2_(THInd)_2_ZrCl_2_ was inactive in CHCl_3_ (entry 9); however, it catalyzed alkene oligomerization in CH_2_Cl_2_ (1-hexene conversion of >99%, entry 8). Thus, the zirconocene ligand structure significantly affects the course of the reaction, and some tested complexes mainly provided the formation of oligomeric products.

### 2.2. NMR Study of Intermediate Structure in MMAO-12-Activated Systems Cp_2_ZrY_2_ (Y = H, Cl)-OAC in Chlorinated Solvents

**Figure 1:**
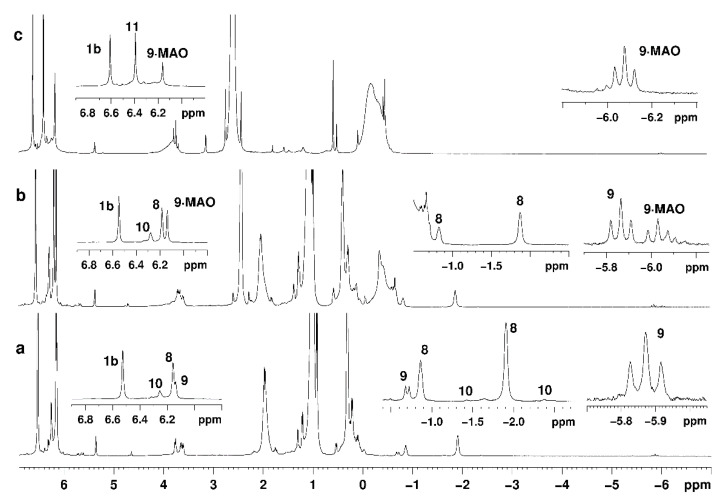
^1^H NMR spectrum of the system [Cp_2_ZrH_2_]_2_-ClAlBu^i^_2_-MMAO-12 in CD_2_Cl_2_ (T = 298 K): (**a**) [Cp_2_ZrH_2_]:[ClAlBu^i^_2_]:[MMAO-12] = 1:2:0; (**b**) [Cp_2_ZrH_2_]:[ClAlBu^i^_2_]:[MMAO-12] = 1:2:5; (**c**) [Cp_2_ZrH_2_]:[ClAlBu^i^_2_] [MMAO-12] = 1:0:7.

The effect of chlorinated solvents (CD_2_Cl_2_ and CDCl_3_) on the structure of the intermediates responsible for the alkene dimerization in the catalytic systems [Cp_2_ZrH_2_]_2_-MMAO-12 was studied by NMR. The addition of MMAO-12 to a solution of [Cp_2_ZrH_2_]_2_ in CD_2_Cl_2_ gives rise to a triplet at −5.95 ppm (J = 17.0 Hz) in the ^1^H NMR spectra (Figure 1c, Figures S21–S23). In the COSY HH spectrum, the triplet correlates with the signal at −0.47 ppm, which is superimposed with the region of resonance lines of methyl group protons of MMAO-12 (−0.82–0.32 ppm). The spectra also exhibited signals for the Cp rings at 6.16 ppm (108.4 ppm in ^13^C NMR spectra), 6.39 ppm (112.8 ppm in ^13^C NMR spectra), and 6.61 ppm (116.2 ppm in ^13^C NMR spectra). The NOESY spectra showed the cross-peaks of the Cp-ring signal at 6.16 ppm with the upfield signals at −5.95 ppm and −0.47 ppm. Relying on these results and the data from previous studies [19,20], these signals were assigned to the biszirconium trihydride complex **9**. The signals at 6.61 ppm and 6.39 ppm correspond to the Cp rings of Cp_2_ZrCl_2_ and Cp_2_ZrMeCl (**11**), respectively [27].

**Scheme 3:**
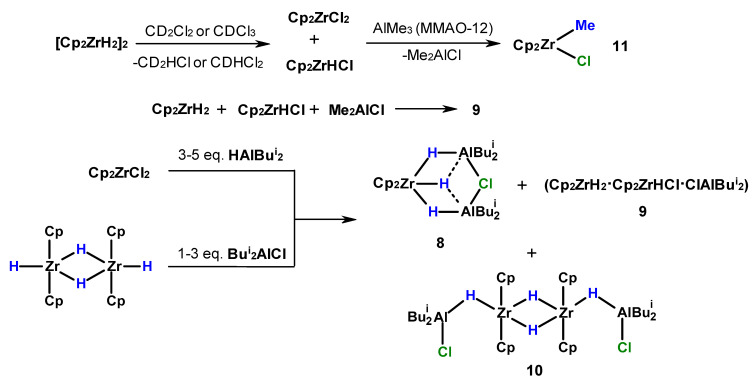
The reaction of Cp_2_ZrCl_2_ and [Cp_2_ZrH_2_]_2_ with OACs and MMAO-12.

As shown in Scheme 3, the system [Cp_2_ZrH_2_]_2_-MMAO-12 in CD_2_Cl_2_ produces complex **9** via in situ formation of Cp_2_ZrHCl and Cp_2_ZrCl_2_ by the reaction of zirconocene dihydride with a chlorine-containing solvent [28]. Residual AlMe_3_ present in the MMAO-12 solution reacts with Cp_2_ZrCl_2_ to give methyl chloride complex **11** and ClAlMe_2_. The reaction of Cp_2_ZrHCl with the starting Cp_2_ZrH_2_ and ClAlMe_2_ makes it possible to selectively obtain complex **9** and then high-molecular-weight associates with MMAO-12 (**9·MAO**), which are active in the alkene dimerization [19,20].

The ^1^H and ^13^C NMR spectra of the system [Cp_2_ZrH_2_]_2_-ClAlBu^i^_2_ (Figure 1a, Figures S14 and S15) in CD_2_Cl_2_ exhibited signals of previously described intermediates [19,23,28,29]: complex **8** (broadened signals of hydride atoms at δ_H_ −0.86 and −1.91 ppm), dimeric complex **10** (broadened signals of hydride atoms at δ_H_ −1.42 and −2.38 ppm), and biszirconium trihydride complex **9** (doublet signal at δ_H_ −0.70 ppm (^2^*J* = 17.0 Hz) and triplet at −5.87 ppm). The addition of MMAO-12 to the equilibrium mixture of the complexes leads to the vanishing of signals of complex **10** from the ^1^H NMR spectrum and the appearance of an additional triplet at −6.01 ppm attributable to the adduct **9·MAO** (Figure 1b, Figures S16–S18).

A similar picture is observed in the ^1^ H and ^13^C NMR spectra of Cp_2_ZrCl_2_-HAlBu^i^_2_ in CD_2_Cl_2_ (Figures S5 and S6). When MMAO-12 is added to the system, the signals of the dimeric complex **10** also disappear, the intensity of the signals of the trihydride **8** decreases, and additional triplets of **9·MAO** appear in the upfield region at −6.21 ÷ −5.92 ppm (Figures S7 and S8).

**Figure 2:**
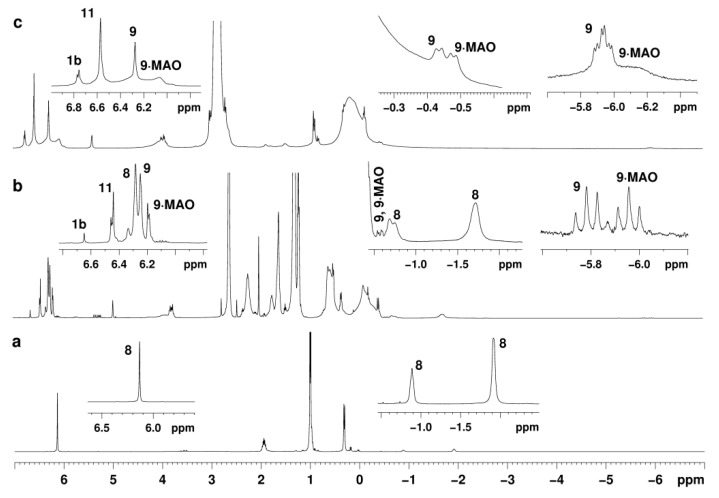
^1^H NMR spectrum of systems Cp_2_ZrCl_2_-HAlBu^i^_2_-MMAO-12 and [Cp_2_ZrH_2_]_2_-MMAO-12 in CDCl_3_ (T = 298 K): (**a**) [Cp_2_ZrCl_2_]:[HAlBu^i^_2_]:[MMAO-12] = 1:4:0; (**b**) [Cp_2_ZrCl_2_]:[HAlBu^i^_2_]:[MMAO-12]:[1-hexene] = 1:2:11:0.1; (**c**) [Cp_2_ZrH_2_]:[MMAO-12] = 1:12.

The reaction of [Cp_2_ZrH_2_]_2_ with MMAO-12 in either CDCl_3_ or CD_2_Cl_2_ gives complexes Cp_2_ZrCl_2_ (δ_Cp_ 6.76 ppm) and Cp_2_ZrMeCl (δ_Cp_ 6.58 ppm) (Figure 2c, Figures S19 and S20). Moreover, the ^1^ H NMR spectrum showed a significant broadening of the signals of the hydride atom at −6.12 ppm and Cp rings of the heavy adduct **9·MAO**, which disappeared from the spectra due to the precipitation of the heavy fraction to the bottom of the NMR tube.

In the reaction of Cp_2_ZrCl_2_ with HAlBu^i^_2_ (1:4) in CDCl_3_, only the Zr,Al-trihydride complex **8** was identified (Figure 2a, Figures S1 and S2). The signals of complexes **9** and **9·MAO** appeared in the ^1^ H NMR spectra after the addition of MMAO-12 and 1-hexene to the system (Figure 2b, Figures S3 and S4). The formation of complexes **8**–**10** was observed in the reaction of zirconocene dihydride with ClAlBu^i^_2_ taken in a 1:2 ratio in CDCl_3_ (Figure S9). The addition of MMAO-12 to this system results in the disappearance of signals of complex **10**, broadening of complex **8** signals, and the appearance of an additional broadened multiplet at −6.03 ppm, corresponding to the heavy adduct **9·MAO** (Figure S11). NMR monitoring of the system’s reaction with 1-hexene showed the consumption of **9·MAO** adduct and the accumulation of the vinylidene dimer (Figures S24 and S25).

Thus, biszirconium complex **9** is readily formed in the systems Cp_2_ZrCl_2_-HAlBu^i^_2_, [Cp_2_ZrH_2_]_2_-MMAO-12, and [Cp_2_ZrH_2_]_2_-ClAlBu^i^_2_ both in CD_2_Cl_2_ and in CDCl_3_. This intermediate reacts with methylaluminoxane to give a heavy adduct, which selectively provides vinylidene dimers of 1-hexene.

### 2.3. DFT Study of the Structure of Biszirconium Complex 9

**Figure 3:**
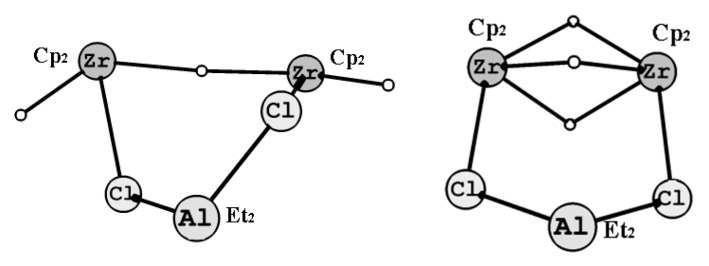
Optimized structures of isomers **9a** and **9c**.

**Scheme 4:**
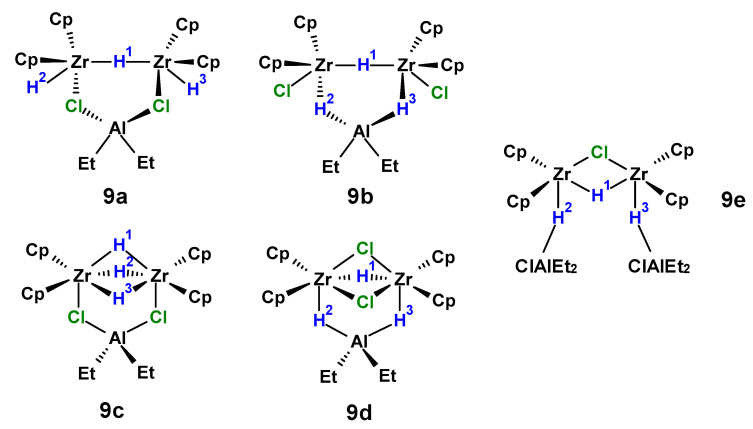
Theoretically calculated structures of isomers of complex **9**.

To refine the structure of complex **9**, probable structures of cyclic isomers **9a**–**9d** were optimized using the PBE/3ζ quantum chemical method [30,31,32]. Their structure is in line with the obtained NMR spectral data on the ratio of the signal intensities of the constituent moieties and the symmetry of the complex (Scheme 4). As follows from Scheme 4, the structures of the complexes significantly differ from one another. For example, in complex **9c**, all three hydrides are located between the zirconium atoms and form a trihydride bridge, while in molecule **9a**, there is only one bridging H atom. If the optimized structures of the two most energetically favorable hydride complexes **9a** and **9c** (Figure 3) are considered in detail, the length of the Zr–H bond varies, which may cause differences in the reactivity of the studied complexes. Thus, the lengths of both Zr–H bonds in the Zr–H–Zr moiety of complex **9a** is 2.09 Å, while the lengths of two terminal Zr–H bonds are *d*_Zr1-H_ = *d*_Zr2-H_ = 1.83 Å. It is comparable with the Zr–H bond length in Cp_2_ZrHCl calculated by the same method (*d*_Zr-H_ = 1.84 Å).

In isomer **9c**, all three hydrogen atoms are inside the biszirconium cage. Meanwhile, the lengths of bridging Zr–H bonds are also increased compared to those in Cp_2_ZrHCl, but are not equivalent to each other: *d*_Zr1-H1_ = *d*_Zr2-H1_ = 2.01 Å, *d*_Zr1-H2_ = *d*_Zr2-H3_ = 1.97 Å, *d*_Zr1-H3_ = *d*_Zr2-H2_ = 1.99 Å. Thus, one hydrogen atom is equidistant from both zirconium atoms, while the other two H atoms, forming an “inner” bridge, are characterized by some displacement towards one of the zirconium atoms. It is quite obvious that structural differences also determine energy differences (Table 4, Table S1).

The most thermodynamically stable complex is **9a**, in which the chlorine atoms are part of the Zr–Cl–Al moieties. Isomer **9b** is higher in energy by 3.3 kcal/mol. The presence of bulky Cl atoms in the inner bridge of the biszirconium cage of structure **9d** makes this complex least thermodynamically stable relative to other compounds.

To identify the structure of the complex observed by NMR spectroscopy, we compared theoretical and experimental NMR chemical shifts of hydride atoms in each of them (Table 5). It was found that in complex **9a**, the hydrogen atom of the Zr–H–Zr moiety is significantly shielded (δH^1^ = −4.2 ppm), while two other hydride atoms experience a de-shielding effect (δH^2^ = δH^3^ = 2.9 ppm). Thus, the difference in chemical shifts between the considered hydrogen atoms is ∆δ = 7.1 ppm, which is in good agreement with the experimental data. A similar trend is also observed for complex **9c**, in which one of the hydride atoms of the inner Zr–H–Zr bridge should be in the upfield region of the ^1^H NMR spectrum. As follows from Table 5, the calculated NMR data for complexes **9b** and **9d** are in poor agreement with the NMR spectral parameters observed. It should be noted for comparison that the calculated chemical shifts of the hydride atoms of the open structure **9e** proposed earlier [23] also do not agree well with the NMR experiment. As a result of a comprehensive analysis of chemical shifts and relative Gibbs energy, the complex **9a** was proposed as the most probable structure.

## 3. Materials and Methods

### 3.1. General Procedures

All operations for organometallic compounds were performed under argon according to the Schlenk technique. Zirconocene **1** was prepared from ZrCl_4_ (99.5%, Merck, Darmstadt, Germany) using the standard procedure [33]. The synthesis of [Cp_2_ZrH_2_]_2_ (**2**) from (**1**) was carried out as described previously [28,34]. The solvents (CHCl_3_, CH_2_Cl_2_, ClCH_2_CH_2_Cl) were distilled from P_2_O_5_ immediately before use. The solvent *o*-Cl_2_C_6_H_4_ (anhydrous, 99%, Merck) was used without further purification. Commercially available Cp_2_HfCl_2_ (98%, Strem, Newburyport, MA, USA), Cp_2_TiCl_2_ (99%, Strem), (C_5_Me_5_)_2_ZrCl_2_ (97%, Acros), *rac*-C_2_H_4_(THInd)_2_ZrCl_2_ (97%, Merck), HAlBu^i^_2_ (99%, Merck), ClAlBu^i^_2_ (97%, Strem), MMAO-12 (7% wt Al in toluene, Merck), (Ph_3_C)[B(C_6_F_5_)_4_] (97%, Abcr, Karlsruhe, Germany), B(C_6_F_5_)_3_ (95%, Merck), and 1-hexene (97%, Fisher Scientific, Pittsburgh, Pennsylvania, USA) were used for the reactions.

CAUTION: pyrophoric nature of aluminum alkyl and hydride compounds requires special safety precautions in their handling.

^1^H and ^13^C NMR spectra were recorded on a Bruker AVANCE−400 spectrometer (400.13 MHz (^1^H), 100.62 MHz (^13^C)) (Bruker, Rheinstetten, Germany). As the solvents and the internal standards, CD_2_Cl_2_ and CDCl_3_ were employed. 1D and 2D NMR spectra (COSY HH, HSQC, HMBC, NOESY) were recorded using standard Bruker pulse sequences.

The products were analyzed using a GC–MS-QP2010 Ultra gas chromatograph–mass spectrometer (Shimadzu, Tokyo, Japan) equipped with the GC−2010 Plus chromatograph (Shimadzu, Tokyo, Japan), TD-20 thermal desorber (Shimadzu, Tokyo, Japan), and an ultrafast quadrupole mass-selective detector (Shimadzu, Tokyo, Japan). Details on the GC–MS analysis of dimers and oligomers are given in the SI.

### 3.2. Reaction of [Cp_2_ZrH_2_]_2_ with ClAlBu^i^_2_, Activators (MMAO-12, (Ph_3_C)[B(C_6_F_5_)_4_] or B(C_6_F_5_)_3_) and 1-Hexene

A flask with a magnetic stirrer was filled under argon with 10 mg (0.045 mmol) [Cp_2_ZrH_2_]_2_ (all ratios are given relative to the monomer), 0.026 mL (0.135 mmol) ClAlBu^i^_2_, 0.58 mL (1.35 mmol) MMAO-12, 2.24 mL or 5,63 mL (17.9 or 45 mmol) 1-hexene, and 2 mL solvent (CHCl_3_, CH_2_Cl_2_, *o*-Cl_2_C_6_H_4_, ClCH_2_CH_2_Cl). For organoboron activators (Ph_3_C)[B(C_6_F_5_)_4_] or B(C_6_F_5_)_3_ the following amounts were used: 10 mg (0.045 mmol) [Cp_2_ZrH_2_]_2_, 0.018–0.036 mL (0.10–0.18 mmol) ClAlBu^i^_2_, 6 mg (0.011 mmol) B(C_6_F_5_)_3_ or 10 mg (0.011 mmol) (Ph_3_C)[B(C_6_F_5_)_4_], 0.6 mL (4.5 mmol) 1-hexene, and 2 mL solvent (CHCl_3_, CH_2_Cl_2_, *o*-Cl_2_C_6_H_4_, ClCH_2_CH_2_Cl). The reaction was carried out with stirring at a temperature of 40 °C. After 5, 10, 15, 30, 60, 90, 120, 180, and 960 min, samples (0.1 mL) were syringed into tubes filled with argon and decomposed with 10% HCl at 0 °C. The products were extracted with CH_2_Cl_2_, and the organic layer was dried over Na_2_SO_4_. The yield of dimers or oligomers was determined by GC/MS.

### 3.3. Reaction of L_2_MCl_2_ (**1a**–**f**) with HAlB ^i^_2_, MMAO-12, (Ph_3_C)[B(C_6_F_5_)_4_] or B(C_6_F_5_)_3_ and 1-Hexene

A flask with a magnetic stirrer was filled under argon with 0.034 mmol (9–15 mg) L_2_MCl_2_, 0.019 mL (0.108 mmol) HAlBu^i^_2_, 0.44 mL (1.02 mmol) MMAO-12, 1.7 or 4.2 mL (13.6 or 34 mmol) 1-hexene, and 2 mL solvents (CHCl_3_, CH_2_Cl_2_, *o*-Cl_2_C_6_H_4_, ClCH_2_CH_2_Cl). For organoboron activators (Ph_3_C)[B(C_6_F_5_)_4_] or B(C_6_F_5_)_3_ the following amounts were used: 0.034 mmol (9–15 mg) L_2_MCl_2_, 0.018–0.036 mL (0.10–0.18 mmol) HAlBu^i^_2_, 5 mg (0.0085 mmol) B(C_6_F_5_)_3_ or 10–16 mg (0.011–0.017 mmol) (Ph_3_C)[B(C_6_F_5_)_4_], 1 or 1.7 mL (8.5 or 13.6 mmol) 1-hexene, and 2 mL solvents (CHCl_3_, CH_2_Cl_2_, o-Cl_2_C_6_H_4_, ClCH_2_CH_2_Cl). The reaction was carried out with stirring at a temperature of 40 °C. After 15, 20, 30, 60, 90, 120, 180, and 960 min, samples (0.1 mL) were syringed into tubes filled with argon and decomposed with 10% HCl or DCl at 0 °C. The products were extracted with CH_2_Cl_2_, and the organic layer was dried over Na_2_SO4. The yields of the products were determined by GC/MS.

### 3.4. NMR Study of the Reaction of [Cp_2_ZrH_2_]_2_ with ClAlR_2_ and MMAO-12

An NMR tube was charged with 0.045 mmol (10 mg) [Cp_2_ZrH_2_]_2_ in an argon-filled glovebox. The tube was cooled to 0 °C, and 0.045–0.2 mmol (8–35.3 mg) ClAlBu^i^_2_ was added dropwise. Then CDCl_3_ or CD_2_Cl_2_ was added. The mixture was stirred, and the formation of complexes **8**–**10** was monitored by NMR at room temperature. Then 0.135–0.9 mmol (60–300 mg) MMAO-12 was added, and the formation of intermediates was monitored by NMR at room temperature (Figures S9–S18).

In the case of the system [Cp_2_ZrH_2_]_2_-MMAO-12, the number of components was the same as described above (Figures S19–S23). The deuterated solvent was added to the NMR tube as the last component.

### 3.5. NMR Study of the Reaction of Cp_2_ZrCl_2_ with HAlBu^i^_2_ and MMAO-12

An NMR tube was charged with 0.034 mmol (10 mg) Cp_2_ZrCl_2_ in an argon-filled glovebox. The tube was cooled to 0 °C, and 0.068–0.136 mmol (10–19.3 mg) HAlBu^i^_2_ was added dropwise. Then CDCl_3_ or CD_2_Cl_2_ was added. The mixture was stirred, and the formation of complexes **8**–**10** was monitored by NMR at room temperature. Then 0.135–0.9 mmol (60–300 mg) MMAO-12 was added, and the formation of intermediates was monitored by NMR at room temperature (Figures S1–S8).

### 3.6. Computational Details

DFT calculations were carried out in Priroda-06 program [32,35]. Geometry optimization, vibrational frequency analysis, and calculation of absolute chemical shielding, entropy, and thermodynamic corrections to the total energy of the compounds were carried out using the Perdew−Burke−Ernzerhof (PBE) functional [30]. PBE functional was used in combination with a 3ζ basis set [31]. The electronic configurations of the molecular systems were described by the orbital basis sets of contracted Gaussian-type functions of size (5s1p)/[3s1p] for H, (11s6p2d)/[6s3p2d] for C, (15s11p2d)/[10s6p2d] for Al and Cl, and (20s16p11d)/[14s11p7d] for Zr, which were used in combination with the density-fitting basis sets of uncontracted Gaussian-type functions of size (5s2p) for H, (10s3p3d1f) for C, (14s3p3d1f1g) for Al and Cl, and (22s5p5d4f4g) for Zr. No symmetry of internal coordinate constraints was applied during optimizations. Thermodynamic parameters were determined at 298.15. Normal-mode vibrational frequency analysis was performed to confirm minima structures. Computations of absolute chemical shielding, σ, were carried out in the GIAO approach [36,37]. Chemical shifts were calculated as δ = σ_TMS_ − σ, where σ_TMS_ was the calculated shielding constant of tetramethylsilane. For comparison, structural and energetic parameters of complexes **9a**–**e** were calculated using Gaussian 09 [38] at the PBE0 level of theory [39] employing the def2-TZVP basis set [40,41] with and without Grimme’s D3(0) empirical dispersion correction (GD3) [42] (Tables S2 and S3). Moreover, the method was successfully employed to calculate chemical shifts of Au hydrides [43]. Calculated chemical shifts obtained for **9a** by two methods was comparable (PBE0/def2-TZVP: δH^1^ = −3.5 ppm, δH^2^ = 3.1 ppm, δH^3^ = 3.1 ppm). Therefore, the PBE/3ζ method was used for the calculation of the chemical shifts of other complexes. As follows from Table S3 (Supporting Information), using the GD3 correction does not lead to significant changes in the energy of the studied complexes. Furthermore, we carried out the study on the solvent effect (CH_2_Cl_2_ and CHCl_3_) using the conductor-like polarizable continuum model (CPCM) [44,45]. The data indicated minor energy changes (Supporting Information, Tables S4 and S5).

The program ChemCraft [46] was used to visualize obtained quantum chemical data. The energy at 0 K, the ZPVE correction, the enthalpy, the Gibbs free energy in gas, the temperature multiplied by the entropy, and Cartesian coordinates for all optimized structures are given in the Supporting Information.

## 4. Conclusions

As a result of studying the catalytic transformations of 1-hexene under the action of Ti group metallocenes, organoaluminum compounds, and activators MMAO-12, B(C_6_F_5_)_3_ or (Ph_3_C)[B(C_6_F_5_)_4_] in chlorinated solvents, it was found that systems based on Zr complexes [Cp_2_ZrH_2_]_2_-ClAlBu^i^_2_-MMAO-12, Cp_2_ZrCl_2_-HAlBu^i^_2_-MMAO-12 in CH_2_Cl_2_ selectively afford dimeric products in high yields. The use of CHCl_3_ as the solvent facilitates the formation of non-classical tetramers of 1-hexene, the products of dimer dimerization.

NMR studies showed that systems Cp_2_ZrCl_2_-HAlBu^i^_2_-MMAO-12, [Cp_2_ZrH_2_]_2_-MMAO-12, and [Cp_2_ZrH_2_]_2_-ClAlBu^i^_2_-MMAO-12 provide the adduct of biszirconium complex (Cp_2_ZrH_2_∙Cp_2_ZrHCl∙ClAlR_2_) with an activator both in CD_2_Cl_2_ and CDCl_3_. The adduct reacts with 1-hexene and produces a vinylidene dimer.

The probable structure of the key biszirconium hydride complex was proposed based on a comparison of experimental and theoretical NMR data and estimation of the thermodynamical stability of the complexes. These studies are necessary to further understand the mechanism of key intermediate activation by methylaluminoxane or organoboron compounds for selective alkene dimerization.

## Figures and Tables

**Table 1:** Catalytic activity and chemoselectivity of systems **A** and **B** in the reaction with 1-hexene.

Entry	Catalytic Systems			[Zr]: [Al]: [Activator]:[1-Hexene]	Solvent	T, °C	Time, min	Alkene Conversion, %		Product Composition, % ^h^				
	Zr Complex	OAC ^a^	Activator						4	5	6			7
											*n* = 1	*n* = 2	*n* = 3	
1 [19]	[Cp_2_ZrH_2_]_2_	ClAlBu^i^_2_	MMAO-12	1:3:30:100	C_6_H_5_CH_3_	40	15	>99 ^b^		86				
2		ClAlBu^i^_2_	MMAO-12	1:3:30:400	CH_2_Cl_2_	40	15	>99		98	1.3			
3							180			98.2	1.8			
4		ClAlBu^i^_2_	MMAO-12	1:3:30:400	CHCl_3_	40	15	92		92				
5							60	>99		98				
6							105			97	1.3	1.6		
7							180		9	12.6	1.4	2		75
8							960		17	-	1.4	2.6		79
9		-	MMAO-12	1:30:400	CHCl_3_	40	30	96	1	91	1			
10					CH_2_Cl_2_			>99	<1	89	8			
11					*o*-Cl_2_C_6_H_4_		60	86	1	73	10	<1		
12							960	93	<1	74	13	3	1	
13					(CH_2_Cl)_2_		60	90		83	5			
14							960	99		91	4	1		
15 [20]		ClAlBu^i^_2_	B(C_6_F_5_)_3_	4:8:1:400	C_6_H_6_	40	90	81		81				
16		-	B(C_6_F_5_)_3_	4:1:400	CHCl_3_	40	960	0						
17					CH_2_Cl_2_		960	0						
18		ClAlBu^i^_2_		4:16:1:400	CHCl_3_		180	42		40	2			
19							960	75		71	4			
20					CH_2_Cl_2_		960	0						
21 [20]		ClAlEt_2_	(Ph_3_C)[B(C_6_F_5_)_4_]	4:8:1:400	C_6_H_6_	40	90	91 ^c^		86				
22		-	(Ph_3_C)[B(C_6_F_5_)_4_]	4:1:400	CHCl_3_	40	960	0						
23					CH_2_Cl_2_		960	0						
24		ClAlBu^i^_2_		4:16:1:400	CHCl_3_		180	44		9	20	-		15
25							960	81		17	36	13		15
26					CH_2_Cl_2_		180	>99		13	18			69
27 [19]	Cp_2_ZrCl_2_	HAlBu^i^_2_	MMAO-12	1:3:30:100	C_6_H_5_CH_3_	40	15	>99 ^d^		91				
28		HAlBu^i^_2_	MMAO-12	1:3:30:400	CH_2_Cl_2_	40	30	98		97	1			
29							60	99		98	1			
30		-		1:30:400	CH_2_Cl_2_	40	180	98		96	-	-		
31							960	99		92	4	<1		
32		HAlBu^i^_2_	MMAO-12	1:3:30:1000	CH_2_Cl_2_	40	30	82		80	2			
33							60	88		77	2			9
34		HAlBu^i^_2_	MMAO-12	1:3:30:400	CHCl_3_	40	30	>99		98	2			
35		HAlBu^i^_2_	MMAO-12	1:3:30:1000	CHCl_3_	40	30	>99		90	7			3
36		HAlBu^i^_2_	MMAO-12	1:3:30:400	CHCl_3_	20	180	>99		97	2			
37		-	MMAO-12	1:30:400	CHCl_3_	40	180	>99	5	3	2			89
38		-	MMAO-12	1:10:400	CHCl_3_	40	960	91		91	<1			
39		-	MMAO-12	1:30:400	*o*-Cl_2_C_6_H_4_	40	30	99		93	4	-		
40							960	99		91	5	1		
41		-	MMAO-12	1:30:400	(CH_2_Cl)_2_	40	30	99		95	2			
42							960	>99		96	2			
43 [20]		HAlBu^i^_2_	B(C_6_F_5_)_3_	4:16:1:1000	C_6_H_6_	40	60	>99 ^e^		93				
44		HAlBu^i^_2_	B(C_6_F_5_)_3_	4:16:1:1000	CH_2_Cl_2_	40	60	>99		99	1			
45					CHCl_3_		60	83		82	1			
46		-	B(C_6_F_5_)_3_	4:1:1000	CH_2_Cl_2_	40	960	0						
47					CHCl_3_	40	960	0						
48 [20]		HAlBu^i^_2_	(Ph_3_C)[B(C_6_F_5_)_4_]	4:16:1:1000	C_6_H_6_	60	90	97 ^f^		67				
49		HAlBu^i^_2_	(Ph_3_C)[B(C_6_F_5_)_4_]	4:16:1:1000	CH_2_Cl_2_	20	180	>99		92	6			2
50							960		1	55	8	3		33
51		HAlBu^i^_2_	(Ph_3_C)[B(C_6_F_5_)_4_]	4:16:1:1000	CH_2_Cl_2_	40	30	>99 ^g^	3	60	6	1		27
52							960			1	2	1	24	43
53		HAlBu^i^_2_	(Ph_3_C)[B(C_6_F_5_)_4_]	4:16:1:1000	CHCl_3_	20	180	>99		76	8	2		13
54						40	180			15	8	11		65
55							960			8	8	12		72
56		-	(Ph_3_C)[B(C_6_F_5_)_4_]	4:1:1000	CH_2_Cl_2_	40	960	0						
57					CHCl_3_		960	0						

^a^ OAC—organoaluminum compound; ^b^ hydrometalation product, 4%; saturated dimer, 2%; unsaturated methylalumination product, 8% [19]; ^c^ hydrometalation product, 5% [20]; ^d^ hydrometalation product, 5%; saturated dimer, 1%; unsaturated methylalumination product, 2%; ^e^ hydrometalation product, 3%; trimers, 3%; ^f^ oligomers, 23%; ^g^ 6-mers (29%) are identified; ^h^ wt % in the reaction mixture, determined by GC–MS of deuterolysis or hydrolysis products (the details of the GC–MS analysis is shown in Supporting Information, Figures S28 and S29).

**Table 2:** Catalytic activity and chemoselectivity of systems Cp_2_MCl_2_-HAlBu^i^_2_-activator (M = Ti, Hf) in the reaction with 1-hexene.

Entry	Catalytic Systems			[Zr]: [Al]: [Activator]:[1-Alkene]	Solvent	T, °C	Time, min	Alkene Conversion, %		Product Composition, % ^d^				
	Zr Complex	OAC	Activator						4	5	6			7
											*n* = 1	*n* = 2	*n* = 3	
1	Cp_2_TiCl_2_^a^	HAlBu^i^_2_	MMAO-12	1:3:30:400	CH_2_Cl_2_	40	60	80		20	34	4		15
2							180	91		17	44	5		20
3					CHCl_3_		60	93		16	36	6	5	28
4							960	>99	5	4	41	11	7	30
5	Cp_2_HfCl_2_	HAlBu^i^_2_	MMAO-12	1:3:30:400	CH_2_Cl_2_	40	120	84	2	53	20	6	2	
6							180	94	2	54	21	8	2	
7							960	96	-	59	22	8	3	4
8			MMAO-12	1:3:30:400	CHCl_3_	40	60	60	-	42	15	3		1
9							120	74 ^b^	-	30	16	4		5
10							960	>99 ^c^	6	17	17	15		9
11	Cp_2_TiCl_2_	HAlBu^i^_2_	(Ph_3_C)[B(C_6_F_5_)_4_]	4:16:1:400	CH_2_Cl_2_	40	960	0						
12					CHCl_3_		960	0						
13	Cp_2_HfCl_2_	HAlBu^i^_2_	(Ph_3_C)[B(C_6_F_5_)_4_]		CH_2_Cl_2_	40	960	0						
14					CHCl_3_		960	0						

^a^ regioselectivity is significantly reduced due to double bond migration; ^b^ byproducts of toluene mono- (13%), di- (4%) and tri- (2%) alkylation with 1-hexene are formed; ^c^ byproducts of toluene mono- (22%), di- (7%), and tri- (4%) alkylation with 1-hexene are formed; ^d^ wt % in the reaction mixture, determined by GC–MS of deuterolysis or hydrolysis products (the details of the GC–MS analysis is shown in Supporting Information).

**Table 3:** Catalytic activity and chemoselectivity of systems L_2_ZrCl_2_-HAlBu^i^_2_-activator in the reaction with 1-hexene.

Entry	Catalytic Systems			[Zr]: [Al]: [activator]:[1-alkene]	Solvent	T, °C	Time, min	Alkene Conversion, %		Product Composition, % ^e^			
	Zr Complex	OAC	Activator						4	5	6		
											*n* = 1	*n* = 2	*n* = 3
1	(C_5_Me_5_)_2_ZrCl_2_	HAlBu^i^_2_	MMAO-12	1:3:30:400	CHCl_3_	40	30	>99 ^a^					
2					CH_2_Cl_2_		30						
3	Ind_2_ZrCl_2_	HAlBu^i^_2_	MMAO-12	1:3:30:400	CH_2_Cl_2_	40	30	19		11	5	2	1
4							180	48		20	19	6	3
5							960	85 ^b^		9	18	18	16
6					CHCl_3_	40	180	>99		13	51	24	12
7							960	>99 ^c^		16	21	19	18
8	*rac*-H_4_C_2_[THInd]_2_ZrCl_2_	HAlBu^i^_2_	MMAO-12	1:3:30:400	CHCl_3_	40	30	>99 ^d^		39	38	16	4
9					CH_2_Cl_2_		960	0					

^a^ 1-hexene is completely consumed for toluene alkylation; ^b^ 6-mers (15%) and 7-mers (9%); ^c^ 6-mers (17%) and 7-mers (9%); ^d^ 6-mers (3%); ^e^ wt % in the reaction mixture, determined by GC–MS of deuterolysis or hydrolysis products (the details of the GC–MS analysis is shown in Supporting Information).

**Table 4:** Relative thermodynamic parameters of isomeric complexes **9.**

Complex	∆E, Hartree	∆E_ZPVE_, Hartree	∆H, kcal/mol	∆G, kcal/mol	T∆S, cal/mol
9a	0.000000	0.000000	0.0	0.0	2475.2
9b	0.013103	0.012948	8.2	9.6	1139.2
9c	0.003022	0.003398	2.4	3.3	1517.2
9d	0.022146	0.023111	14.4	16.9	0.0

**Table 5:** Calculated and experimental chemical shifts of isomeric complexes **9a**–**d** (numbering according to Scheme 4).

Complex	δ(H ^1^), ppm	δ(H ^2^), ppm	δ(H ^3^), ppm	δ(Cp), ppm
9a	−4.2	2.9	2.9	5.9
9b	−2.3	1.8	1.8	6.2
9c	−3.6	3.7	3.7	6.2
9d	0.7	0.8	0.8	6.0
9e	−1.6	−0.4	−0.4	6.1
9 (experimental)	−5.9	−0.7	−0.7	6.1

## Data Availability

Data is contained within the article.

## Supplementary Materials

Supplementary Materials are available online. Figures S1–S27: NMR spectra of catalytic systems, Figures S28 and S29: Examples of GC-MS analysis of dimers and oligomers, Tables S1–S5: Calculated thermodynamic parameters of isomeric complexes **9a–d**.

## Sample Availability

Samples of the compounds are not available from the authors.
