# Supplementary material for: Ti Group Metallocene-Catalyzed Synthesis of 1-Hexene Dimers and Tetramers
Source: Molecules. 2021 May 8;26(9):2775. doi: 10.3390/molecules26092775 (PMC8125888; doi:10.3390/molecules26092775)
Supplement: Supplementary file 1 [file molecules-26-02775-s001.zip › molecules-1198670-supplementary.pdf]

molecules

MDPI

Supporting Information

Ti group metallocene-catalyzed synthesis of 1-hexene dimers and tetramers

Pavel Kovyazin, Almira Bikmeeva, Denis Islamov, Vasiliy Yanybin, Tatyana Tyumkina and Lyudmila Parfenova *

Institute of Petrochemistry and Catalysis of Russian Academy of Sciences, Prospekt Oktyabrya, 141, 450075 Ufa, Russia; kpv38@mail.ru (P.V.K.); almira.bikmeeva@gmail.com (A.K.B.); islamov19@gmail.com (D.N.I.); vyanybin@gmail.com (V.M.Y.); ttvnmr@gmail.com (T.V.T.)
* Correspondence: luda_parfenova@ipc-ras.ru; Tel.: +7-347-284-3527

Figure S1. ^{1}H NMR of system Cp_2ZrCl_2−$HAlBu^i_2$ (1:4) in $CDCl_3$ (T= 299 K).................. 3

Figure S2. ^{13}C NMR of system Cp_2ZrCl_2−$HAlBu^i_2$ (1:4) in $CDCl_3$ (T= 299 K). 3

Figure S3. ^{1}H NMR of system Cp_2ZrCl_2−$HAlBu^i_2$–MMAO-12– 1-hexene (1:4:8:0.1) in $CDCl_3$ (T= 299 K)... 4

Figure S4. ^{13}C NMR of system Cp_2ZrCl_2−$HAlBu^i_2$–MMAO-12– 1-hexene (1:4:8:0.1) in $CDCl_3$ (T= 299 K)... 5

Figure S5. ^{1}H NMR of system Cp_2ZrCl_2−$HAlBu^i_2$ (1:2) in CD_2Cl_2 (T= 298.6 K)............ 6

Figure S6. ^{13}C NMR of system Cp_2ZrCl_2−$HAlBu^i_2$ (1:2) in CD_2Cl_2 (T= 298.6 K)............ 7

Figure S7. ^{1}H NMR of system Cp_2ZrCl_2−$HAlBu^i_2$–MMAO-12 (1:2:5) in CD_2Cl_2 (T= 298.6 K).8

Figure S8. ^{13}C NMR of system Cp_2ZrCl_2−$HAlBu^i_2$–MMAO-12 (1:2:5) in CD_2Cl_2 (T= 299.3 K). 9

Figure S9. ^{1}H NMR of system $[Cp_2ZrH_2]_2$−$ClAlBu^i_2$ (1:2) in $CDCl_3$ (T= 298.7 K). 10

Figure S10. ^{13}C NMR of system $[Cp_2ZrH_2]_2$−$ClAlBu^i_2$ (1:2) in $CDCl_3$ (T= 299.1 K). 11

Figure S11. ^{1}H NMR of system $[Cp_2ZrH_2]_2$−$ClAlBu^i_2$–MMAO-12 (1:2:11) in $CDCl_3$ (T= 299 K). 11

Figure S12. ^{13}C NMR of system $[Cp_2ZrH_2]_2$−$ClAlBu^i_2$–MMAO-12 (1:2:11) in $CDCl_3$ (T= 299.2 K). 12

Figure S13. COSY HH of system $[Cp_2ZrH_2]_2$−$ClAlBu^i_2$–MMAO-12 (1:2:11) in $CDCl_3$ (T= 298.7 K). 13

Figure S14. ^{1}H NMR of system $[Cp_2ZrH_2]_2$−$ClAlBu^i_2$ (1:2) in CD_2Cl_2 (T= 299.1 K)....... 14

Figure S15. ^{13}C NMR of system $[Cp_2ZrH_2]_2$−$ClAlBu^i_2$ (1:2) in CD_2Cl_2 (T= 299.7 K). 15

Figure S16. ^{1}H NMR of system $[Cp_2ZrH_2]_2$−$ClAlBu^i_2$–MMAO-12 (1:2:5) in CD_2Cl_2 (T= 299.1 K). 15

Figure S17. ^{13}C NMR of system $[Cp_2ZrH_2]_2$−$ClAlBu^i_2$–MMAO-12 (1:2:5) in CD_2Cl_2 (T= 299.5 K). 16

Figure S18. COSY HH NMR of system $[Cp_2ZrH_2]_2$−$ClAlBu^i_2$–MMAO-12 (1:2:5) in CD_2Cl_2 (T= 298.7 K). 17

Figure S19. ^{13}C of system $[Cp_2ZrH_2]_2$ – MMAO-12 (1:12) in $CDCl_3$ (T= 296.1 K). 18

Figure S20. COSY HH NMR of of system $[Cp_2ZrH_2]_2$–MMAO-12 (1:12) in $CDCl_3$ (T= 296 K). 19

Figure S21. ^1H of system $[Cp_2ZrH_2]_2$ – MMAO-12 (1:7) in CD_2Cl_2 (T= 296.3 K). 20

Figure S22. ^{13}C NMR of system $[Cp_2ZrH_2]_2$ – MMAO-12 (1:7) in CD_2Cl_2 (T= 296.6 K). 20

Figure S23. COSY HH of system $[Cp_2ZrH_2]_2$ – MMAO-12 (1:7) in CD_2Cl_2 (T= 296 K). 21

Figure S24. ^1H NMR of dimer **5** obtained in the system Cp_2ZrCl_2−$HAlBu^i_2$–MMAO-12, [Zr]:[Al]:[MMAO-12]:[1-alkene]=1:4:8:1 in $CDCl_3$ (T= 298.7 K), 20 min. 22

Figure S25. ^{13}C NMR of dimer **5** obtained in the system Cp_2ZrCl_2−$HAlBu^i_2$–MMAO-12, [Zr]:[Al]:[MMAO-12]:[1-alkene]=1:4:8:1 in $CDCl_3$ (T= 298.7 K), 20 min. 23

Figure S26. ^1H NMR of tetramers **7** obtained in the system Cp_2ZrCl_2–$HAlBu^i_2$–$(Ph_3C)[B(C_6F_5)_4]$, [Zr]:[Al]:[B]:[1-alkene]= 4:16:1:1000, $CHCl_3$, 40°C, 16 h. 24

Figure S27. ^{13}C NMR of tetramers **7** obtained in the system Cp_2ZrCl_2–$HAlBu^i_2$–$(Ph_3C)[B(C_6F_5)_4]$, [Zr]:[Al]:[B]:[1-alkene]= 4:16:1:1000, $CHCl_3$, 40°C, 16 h. 25

GC-MS analysis of products 25

Figure S28. Example of GC-MS analysis of dimers and oligomers obtained in the system $[Cp_2ZrH_2]_2$–$ClAlBu^i_2$–MMAO-12, [Zr]:[Al]:[MMAO-12]:[1-alkene]= 1:3:30:1000, CH_2Cl_2, 40°C, 16 h. 26

Figure S29. Example of GC-MS analysis of dimers and oligomers obtained in the system Cp_2ZrCl_2–$HAlBu^i_2$–$(Ph_3C)[B(C_6F_5)_4]$, [Zr]:[Al]:[B]:[1-alkene]= 4:16:1:1000, $CHCl_3$, 40°C, 16 h. 27

Table S1. The energy at 0K, the ZPVE correction, the enthalpy, the Gibbs free energy in gas, the temperature multiplied by the entropy for complexes **9a-e**, calculated at PBE/3ζ level (298.15 K). 29

Table S2. Relative thermodynamic parameters of isomeric complexes **9a-d**, calculated at PBE0/def2TZVP level (298.15 K). 29

Table S3. Relative thermodynamic parameters of isomeric complexes **9a-d**, calculated at PBE0-GD3/def2TZVP level (298.15 K). 29

Table S4. Relative thermodynamic parameters isomeric complexes **9a-d** in $CHCl_3$ solution, calculated at PBE0/def2TZVP level (298.15 K). 29

Table S5. Relative thermodynamic parameters of isomeric complexes **9a-d** in CH_2Cl_2 solution, calculated at PBE0/def2TZVP level (298.15 K). 29

Atomic coordinates of the complexes **9a-e** 31

Figure S1. ^1H NMR of system Cp_2ZrCl_2−$HAlBu^i_2$ (1:4) in $CDCl_3$ (T= 299 K).

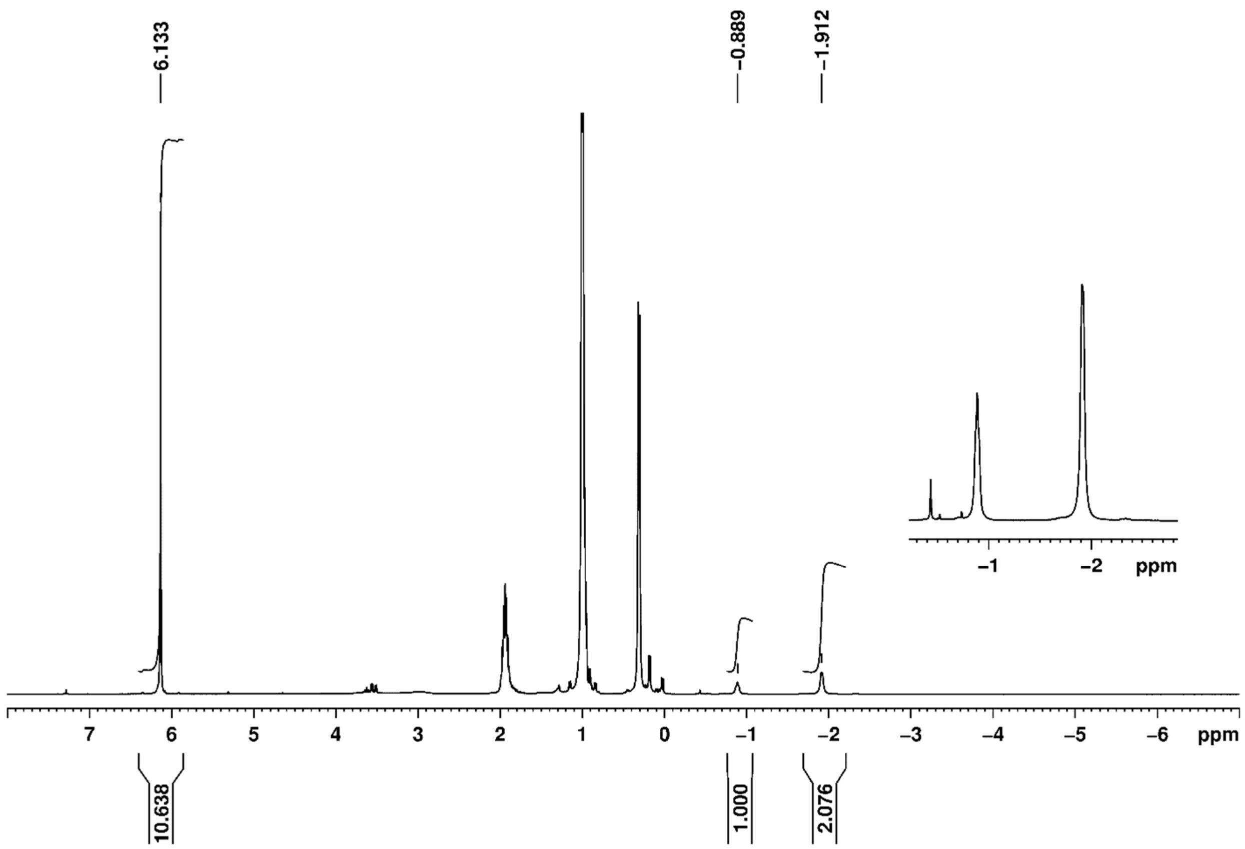

Figure S2. ^{13}C NMR of system Cp_2ZrCl_2−$HAlBu^i_2$ (1:4) in $CDCl_3$ (T= 299 K).

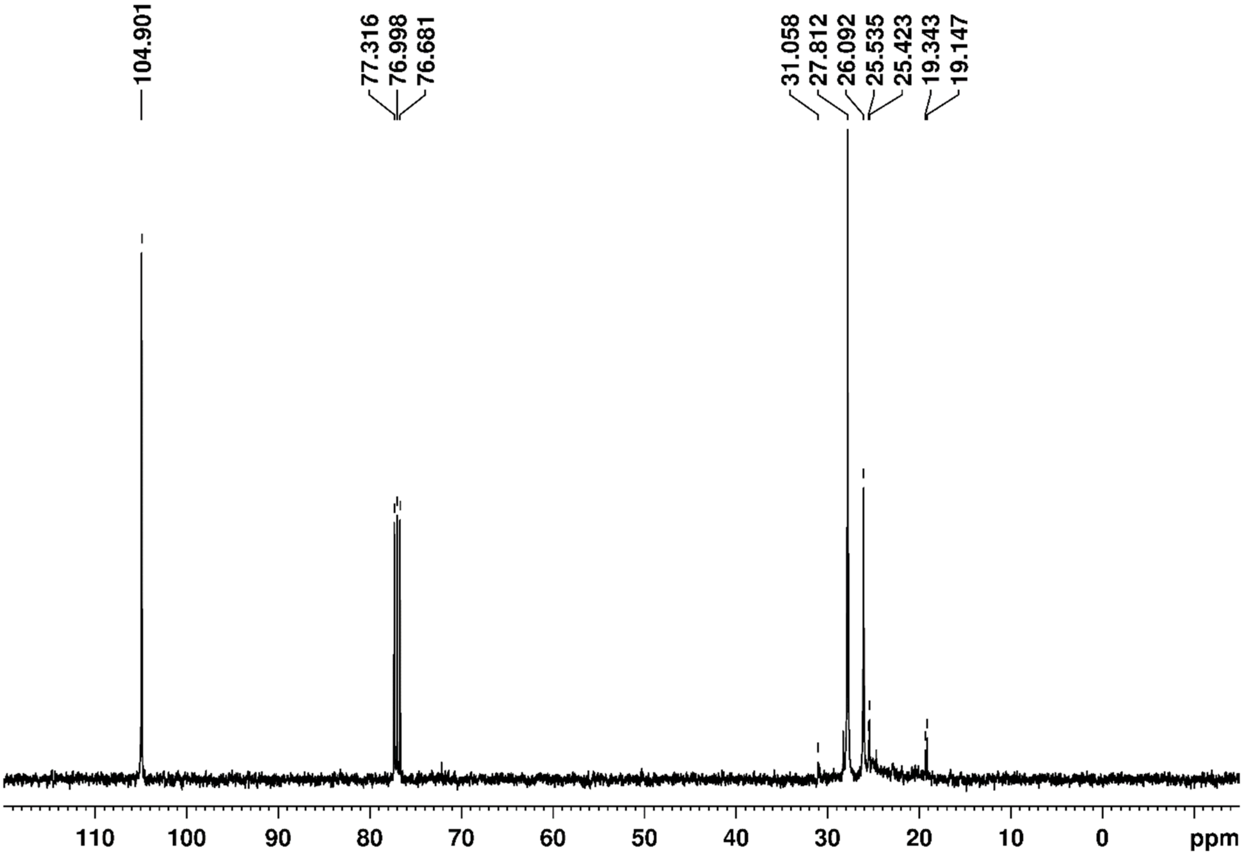

Figure S3. ^{1}H NMR of system Cp_2ZrCl_2−$HAlBu^i_2$–MMAO-12– 1-hexene (1:4:8:0.1) in $CDCl_3$ (T= 299 K).

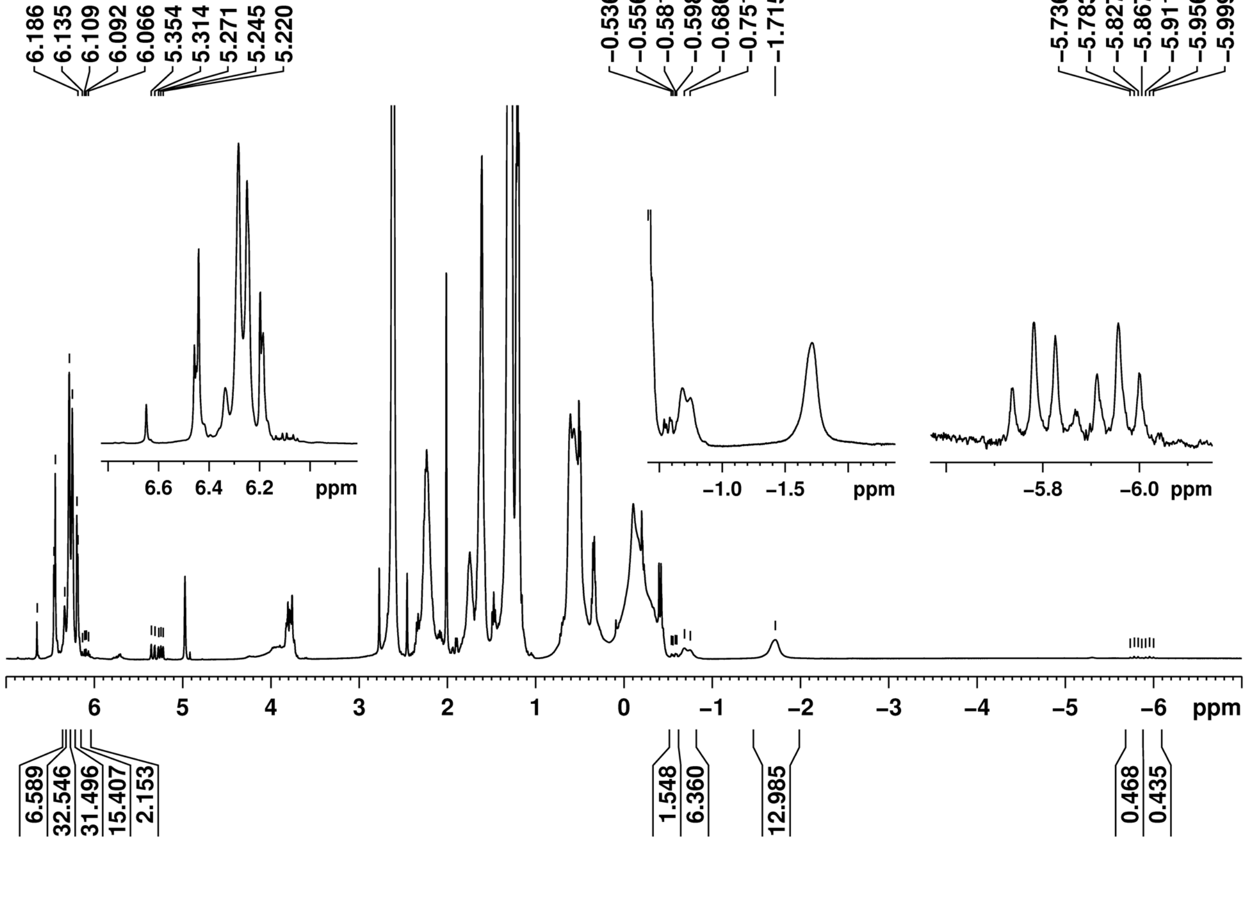

Figure S4. ^{13}C NMR of system Cp_2ZrCl_2−$HAlBu^i_2$–MMAO-12– 1-hexene (1:4:8:0.1) in $CDCl_3$ (T= 299 K).

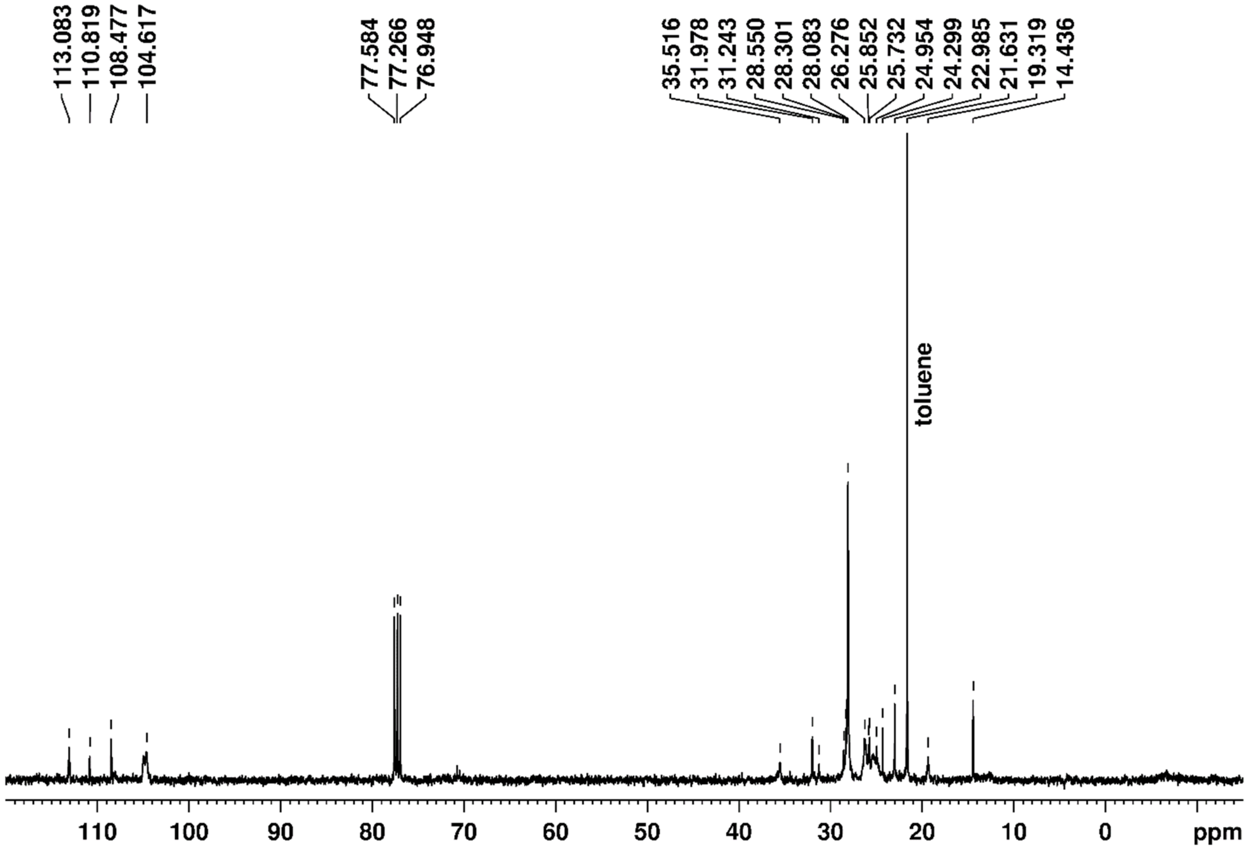

Figure S5. ^{1}H NMR of system Cp_2ZrCl_2−$HAlBu^i_2$ (1:2) in CD_2Cl_2 (T= 298.6 K).

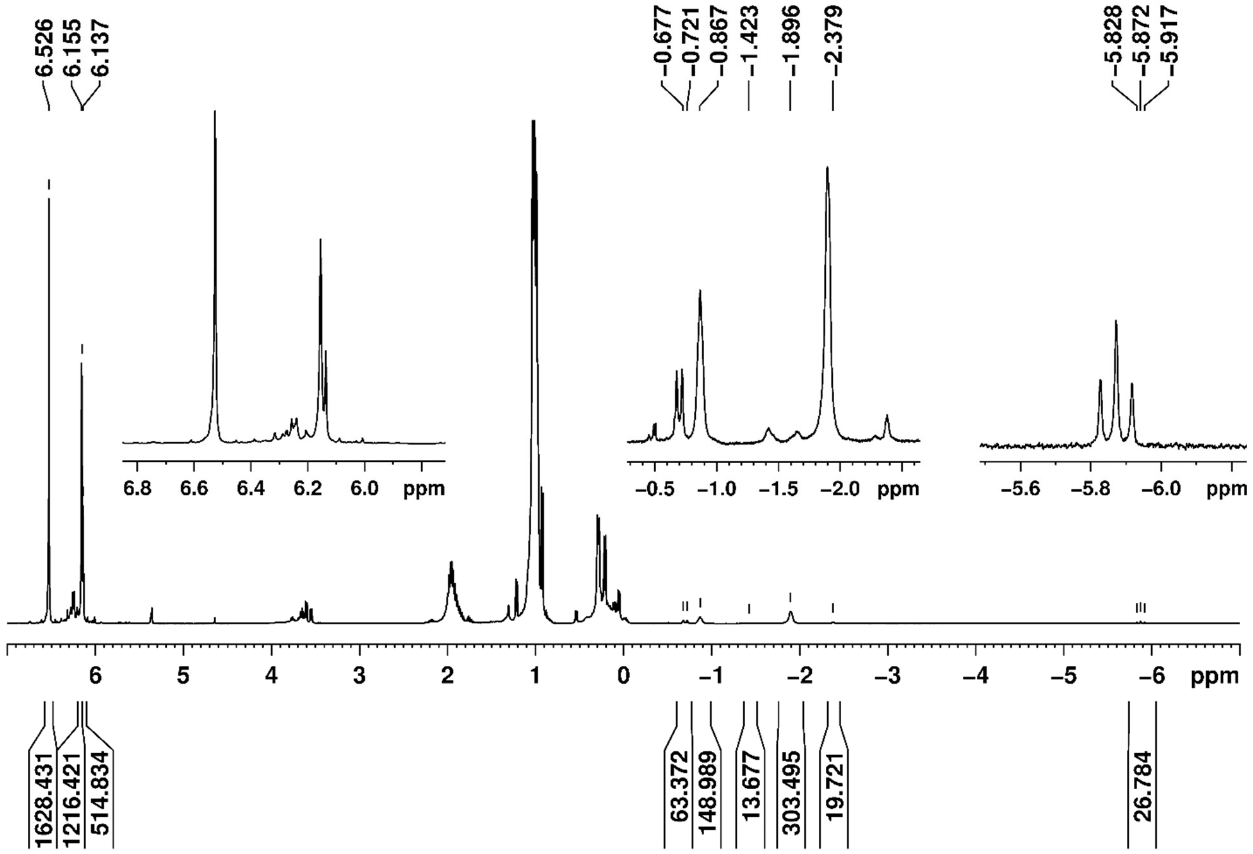

Figure S6. ^{13}C NMR of system Cp_2ZrCl_2−$HAlBu^i_2$ (1:2) in CD_2Cl_2 (T= 298.6 K).

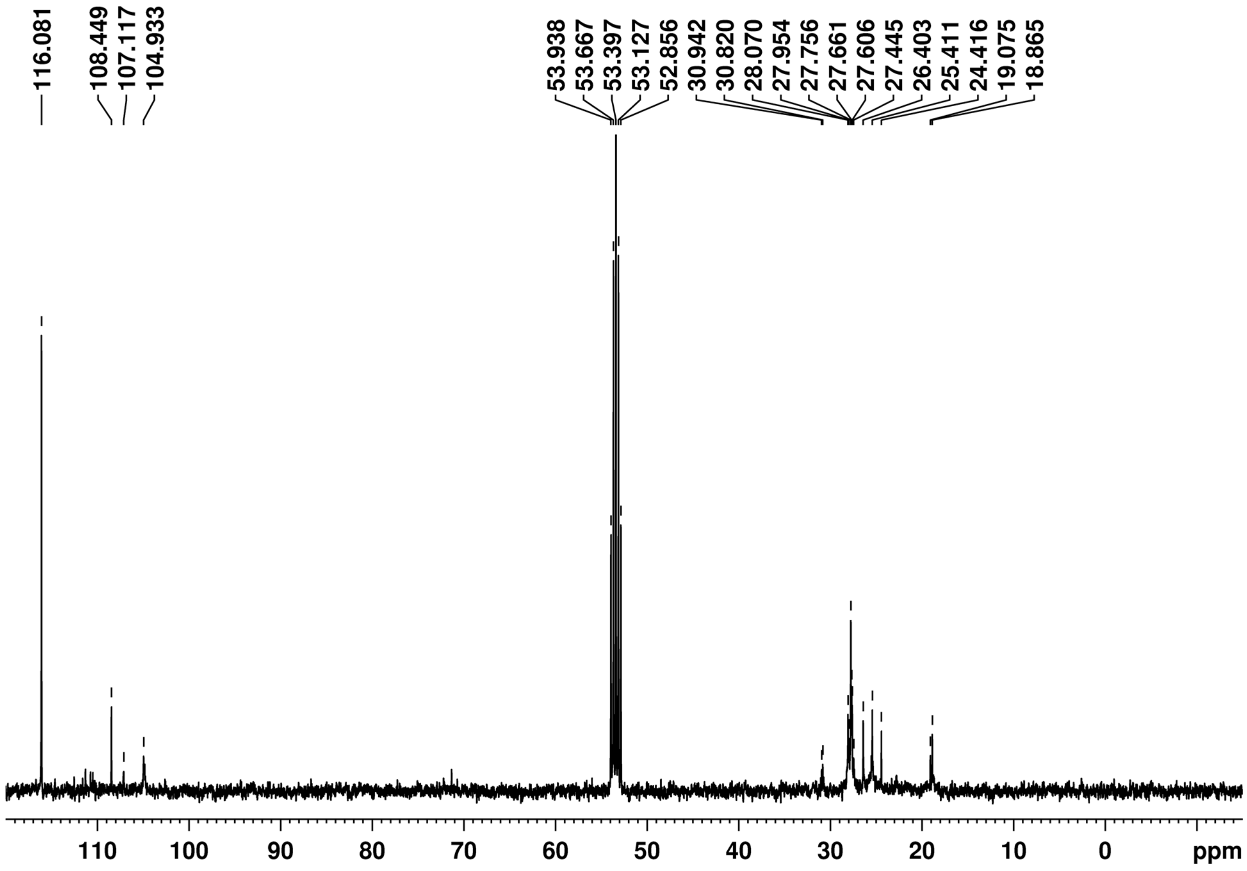

Figure S7. ^{1}H NMR of system Cp_2ZrCl_2–$HAlBu^i_2$–MMAO-12 (1:2:5) in CD_2Cl_2 (T= 298.6 K).

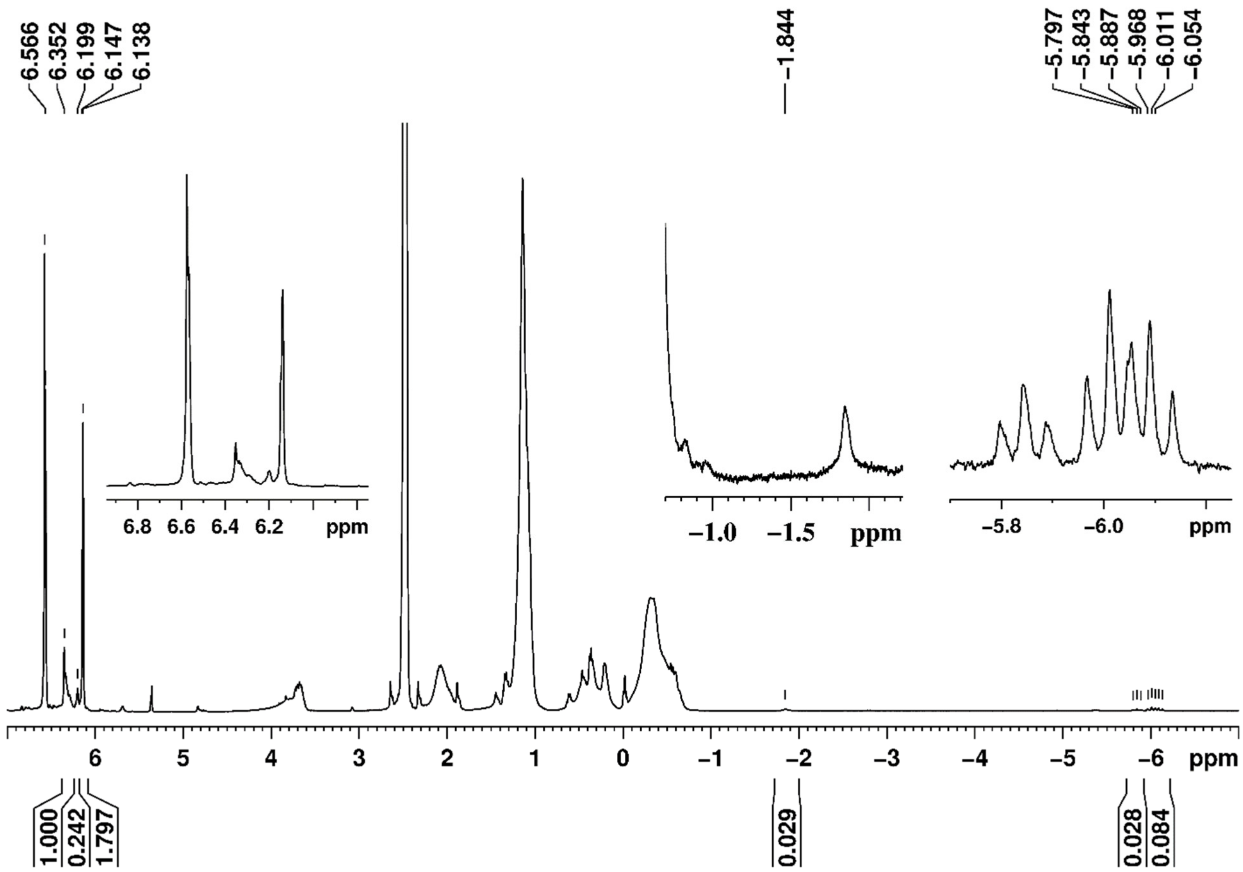

Figure S8. ^{13}C NMR of system Cp_2ZrCl_2−$HAlBu^i_2$–MMAO-12 (1:2:5) in CD_2Cl_2 (T= 299.3 K).

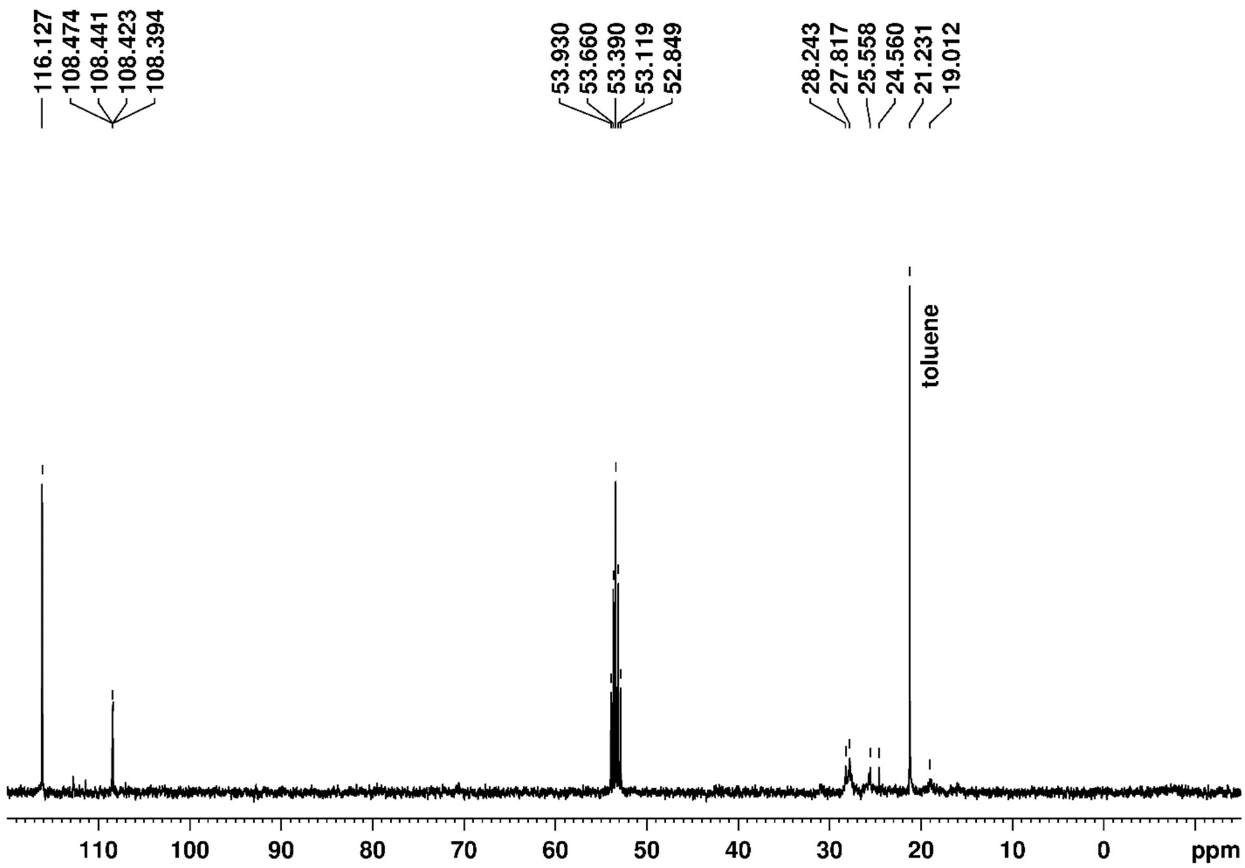

Figure S9. ^{1}H NMR of system $[Cp_2ZrH_2]_2$−$ClAlBu^i_2$ (1:2) in $CDCl_3$ (T= 298.7 K).

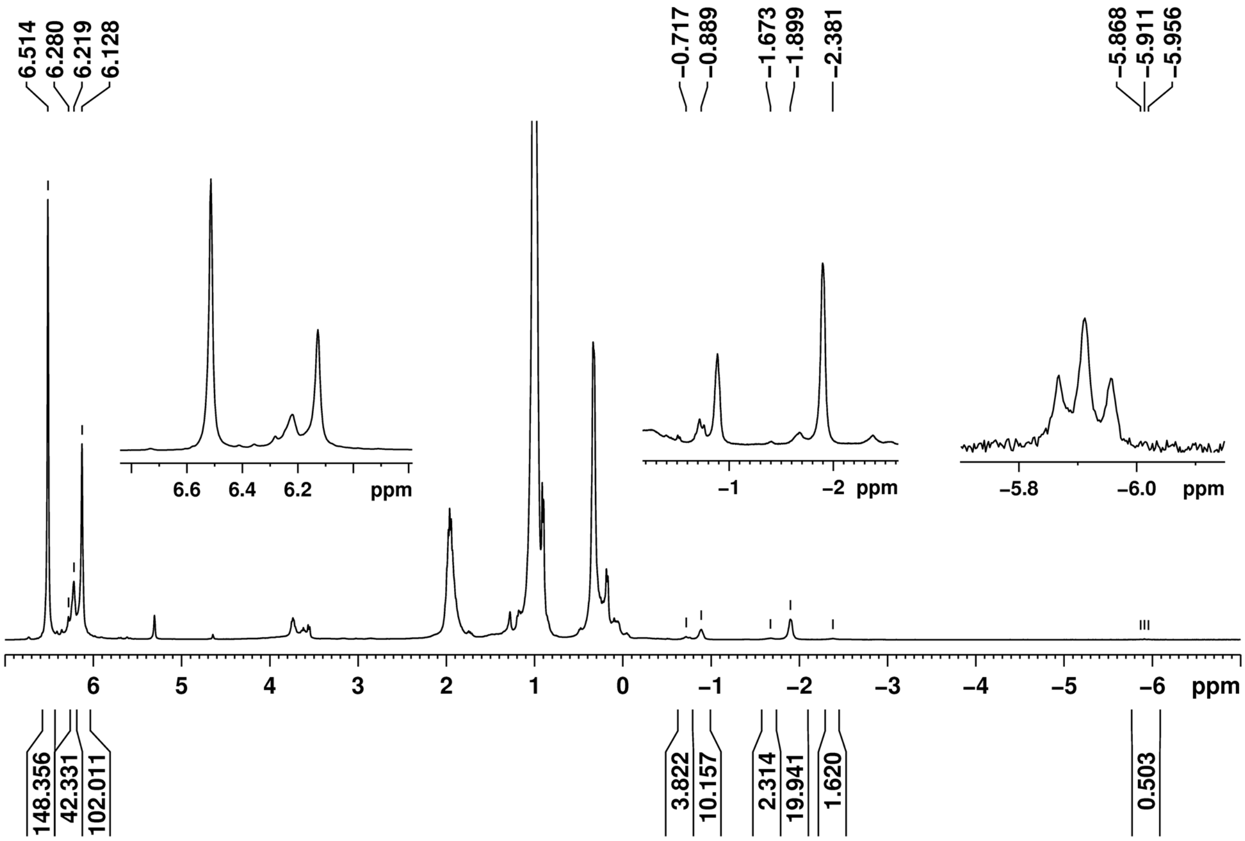

Figure S10. ^{13}C NMR of system $[Cp_2ZrH_2]_2$−$ClAlBu^i_2$ (1:2) in $CDCl_3$ (T= 299.1 K).

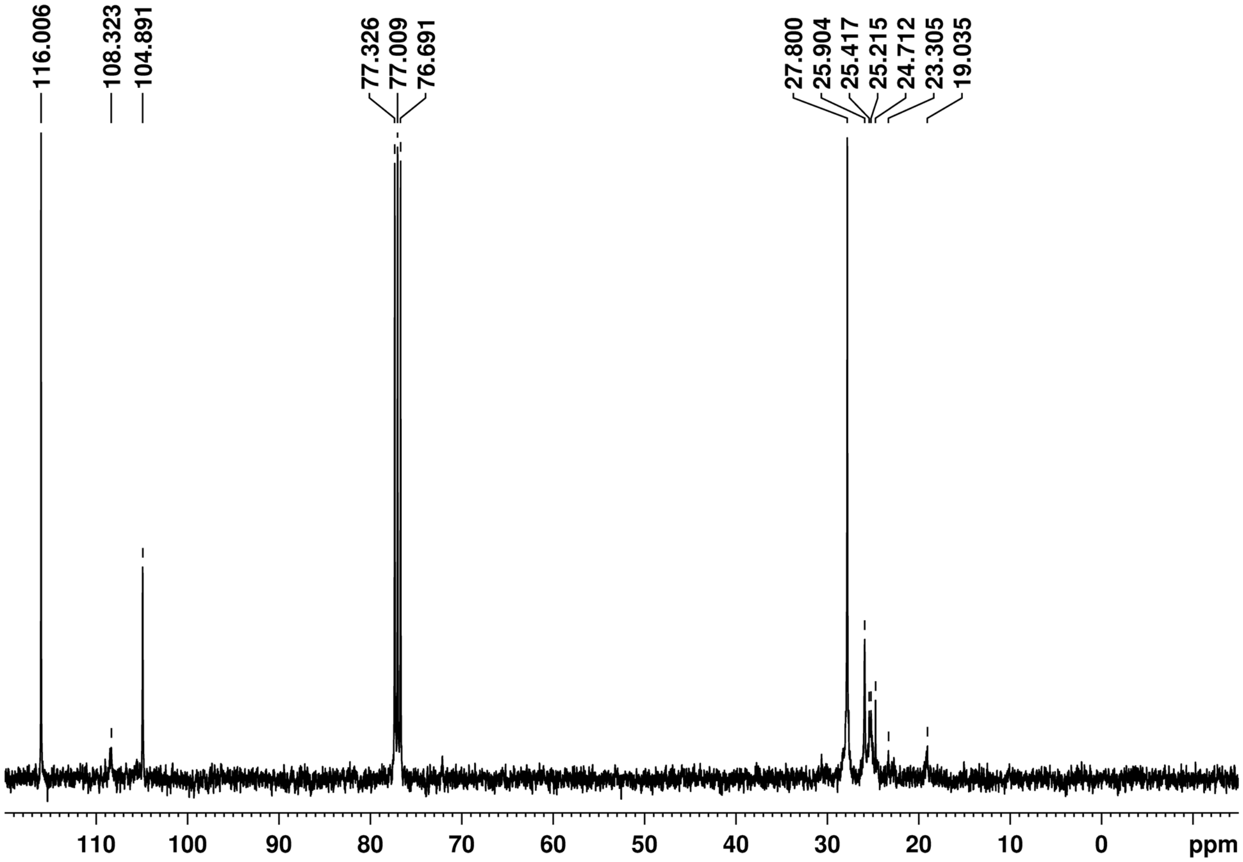

Figure S11. ^{1}H NMR of system $[Cp_2ZrH_2]_2$−$ClAlBu^i_2$–MMAO-12 (1:2:11) in $CDCl_3$ (T= 299 K).

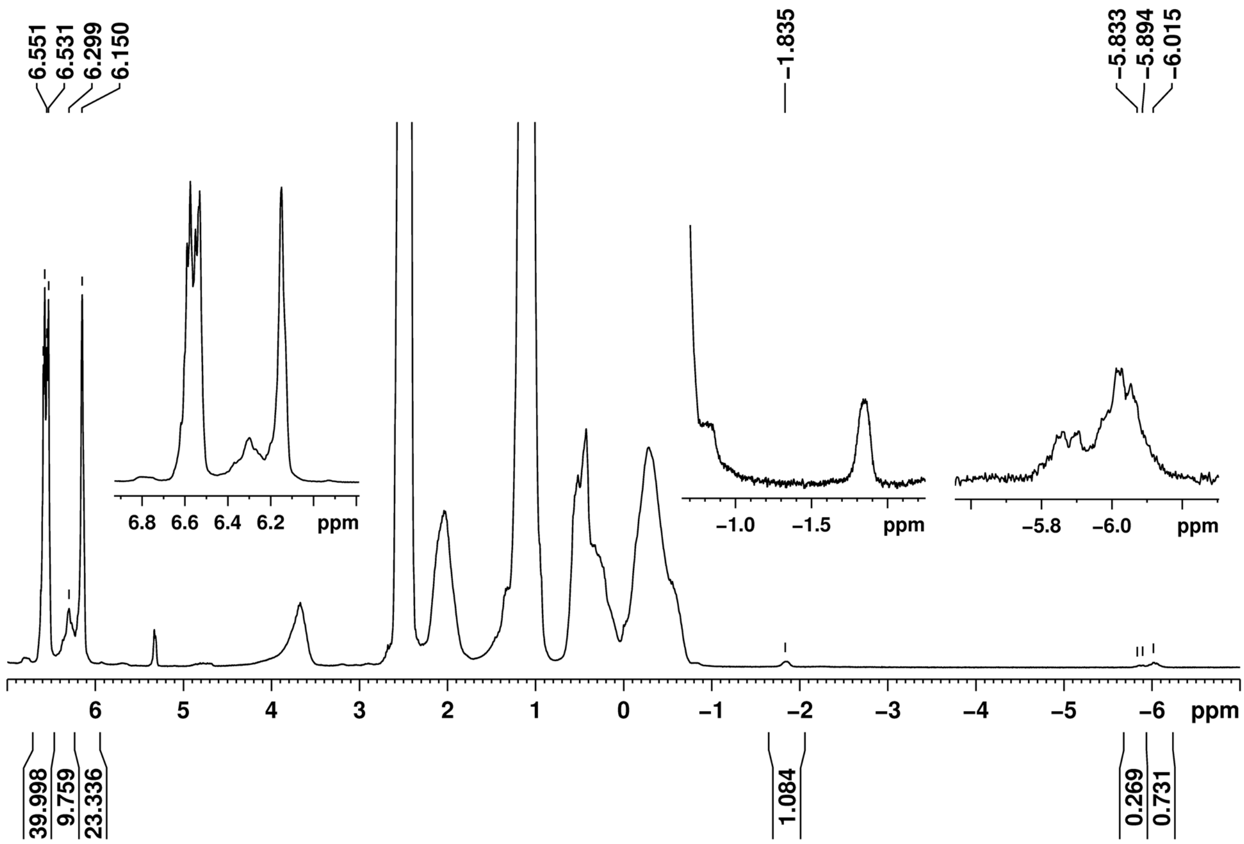

Figure S12. ^{13}C NMR of system $[Cp_2ZrH_2]_2$−$ClAlBu^i_2$–MMAO-12 (1:2:11) in $CDCl_3$ (T= 299.2 K).

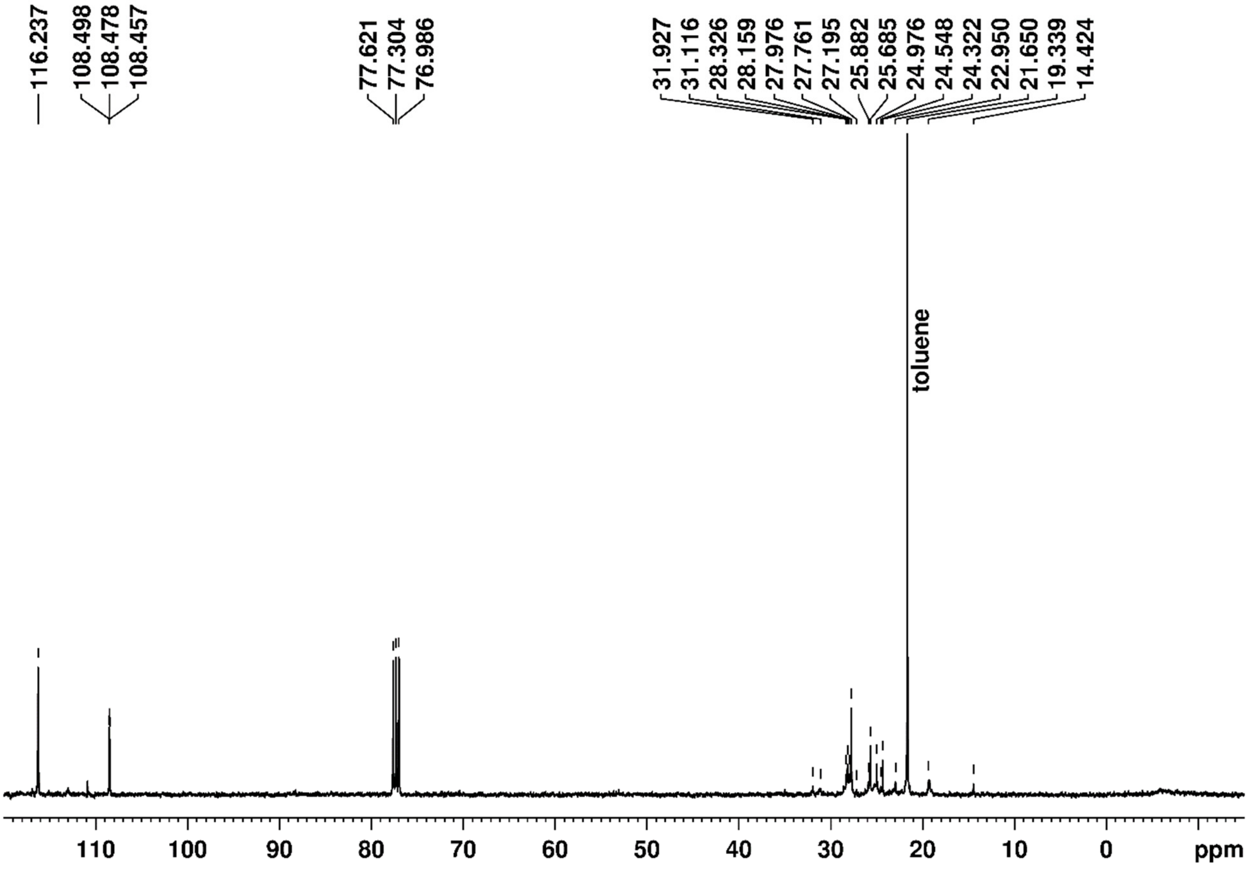

Figure S13. COSY HH of system $[Cp_2ZrH_2]_2$–$ClAlBu^i_2$–MMAO-12 (1:2:11) in $CDCl_3$ (T= 298.7 K).

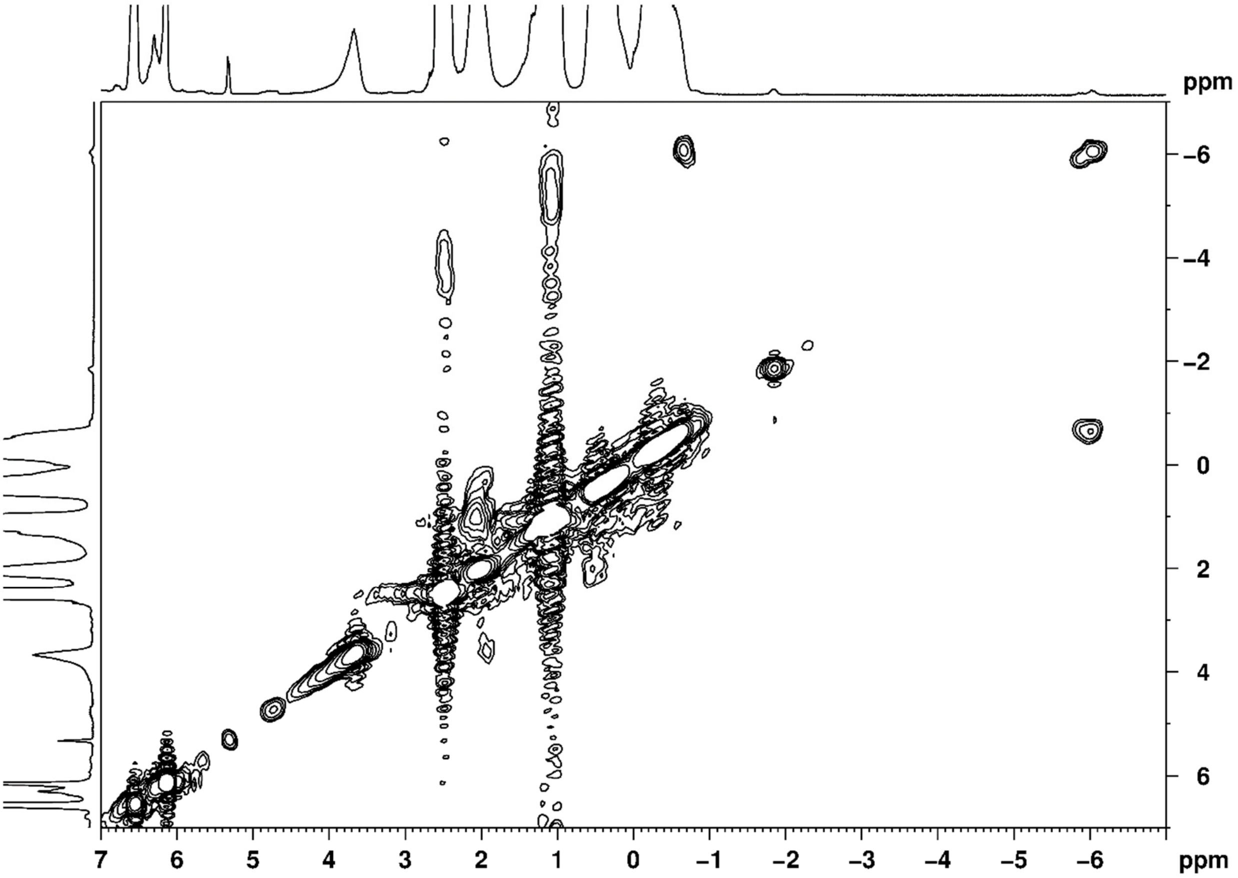

Figure S14. ^{1}H NMR of system $[Cp_2ZrH_2]_2$−$ClAlBu^{i}_2$ (1:2) in CD_2Cl_2 (T= 299.1 K).

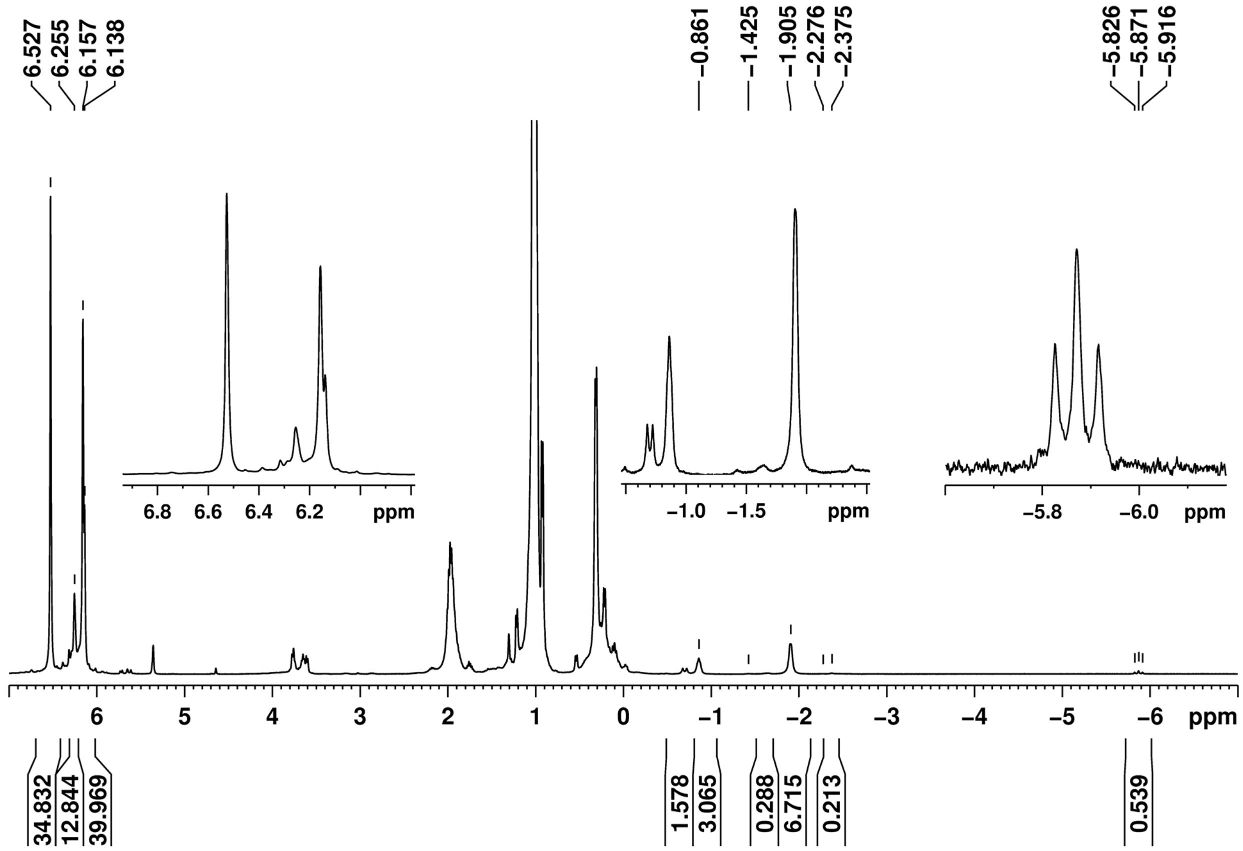

Figure S15. ^{13}C NMR of system $[Cp_2ZrH_2]_2$−$ClAlBu^i_2$ (1:2) in CD_2Cl_2 (T= 299.7 K).

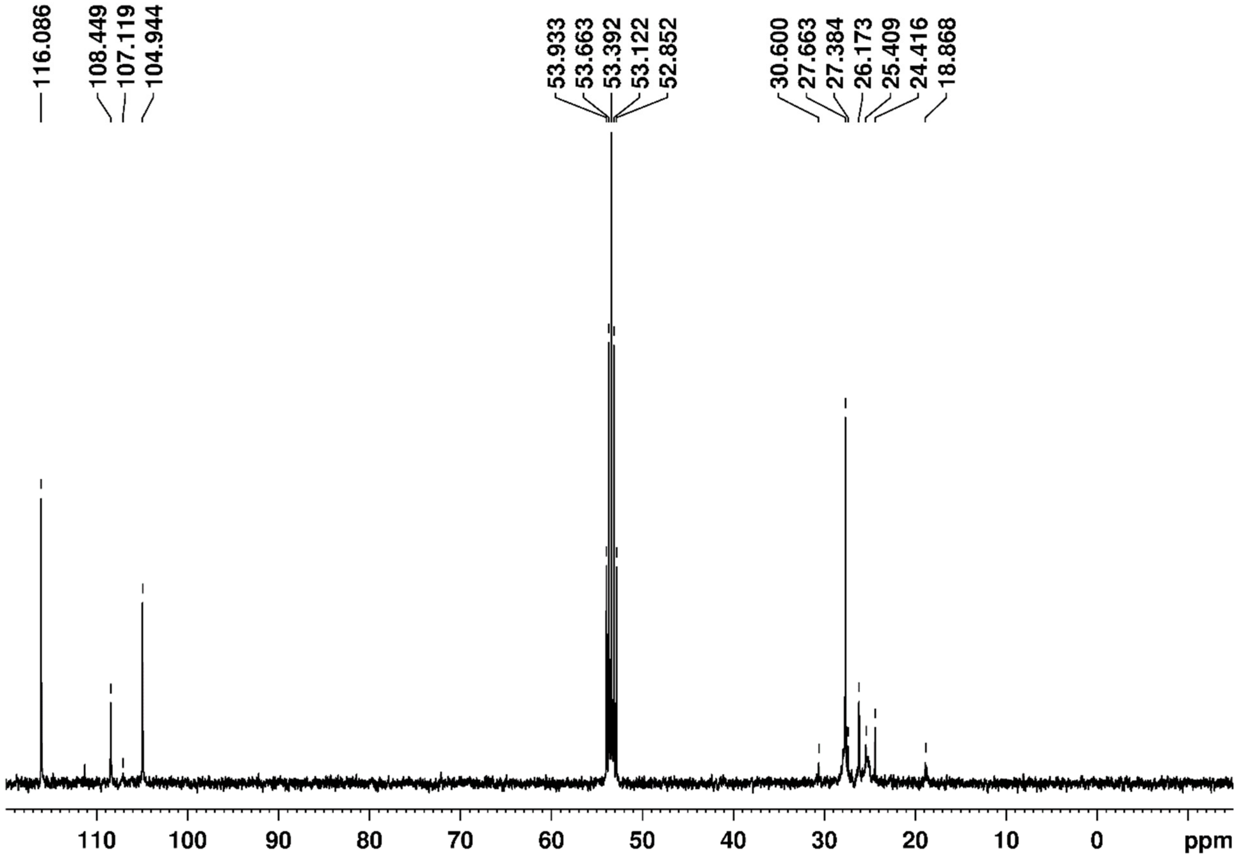

Figure S16. ^1H NMR of system $[Cp_2ZrH_2]_2$−$ClAlBu^i_2$–MMAO-12 (1:2:5) in CD_2Cl_2 (T= 299.1 K).

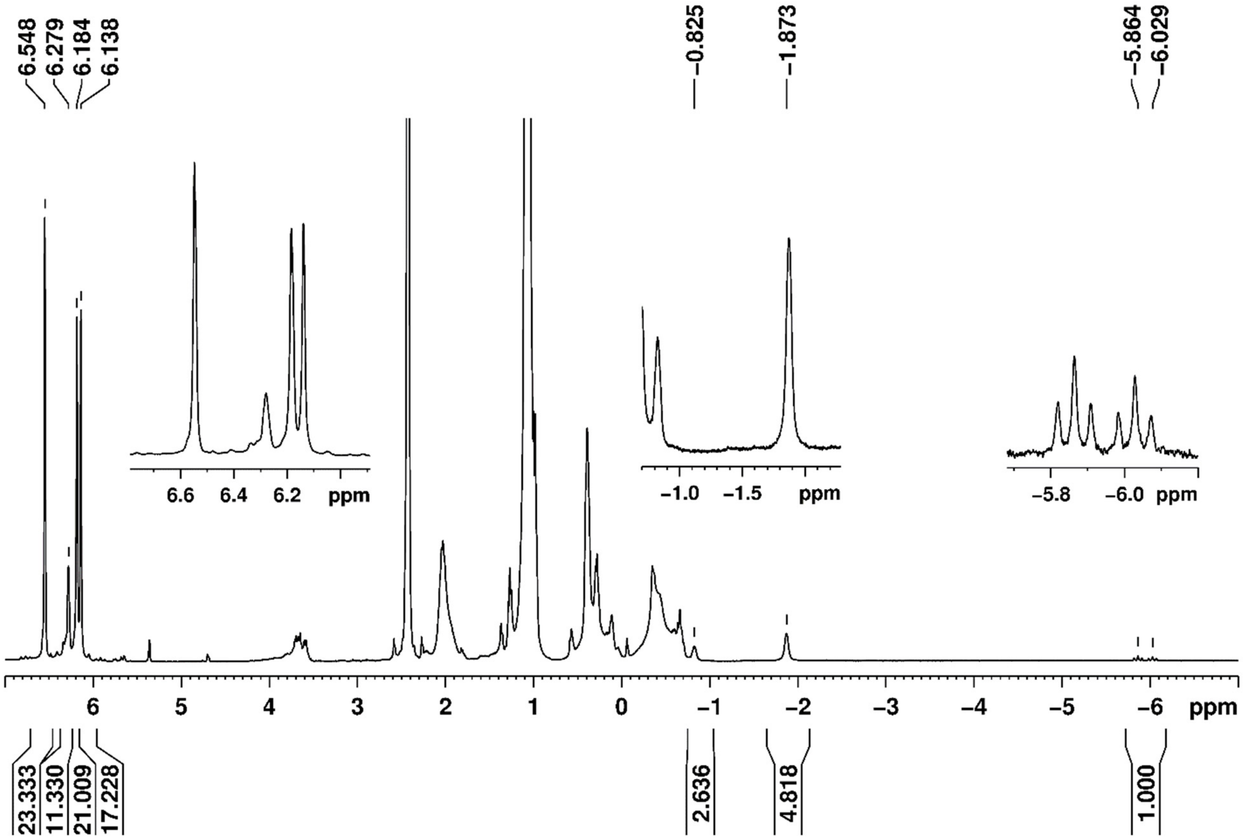

Figure S17. ^{13}C NMR of system $[Cp_2ZrH_2]_2$−$ClAlBu^i_2$–MMAO-12 (1:2:5) in CD_2Cl_2 (T= 299.5 K).

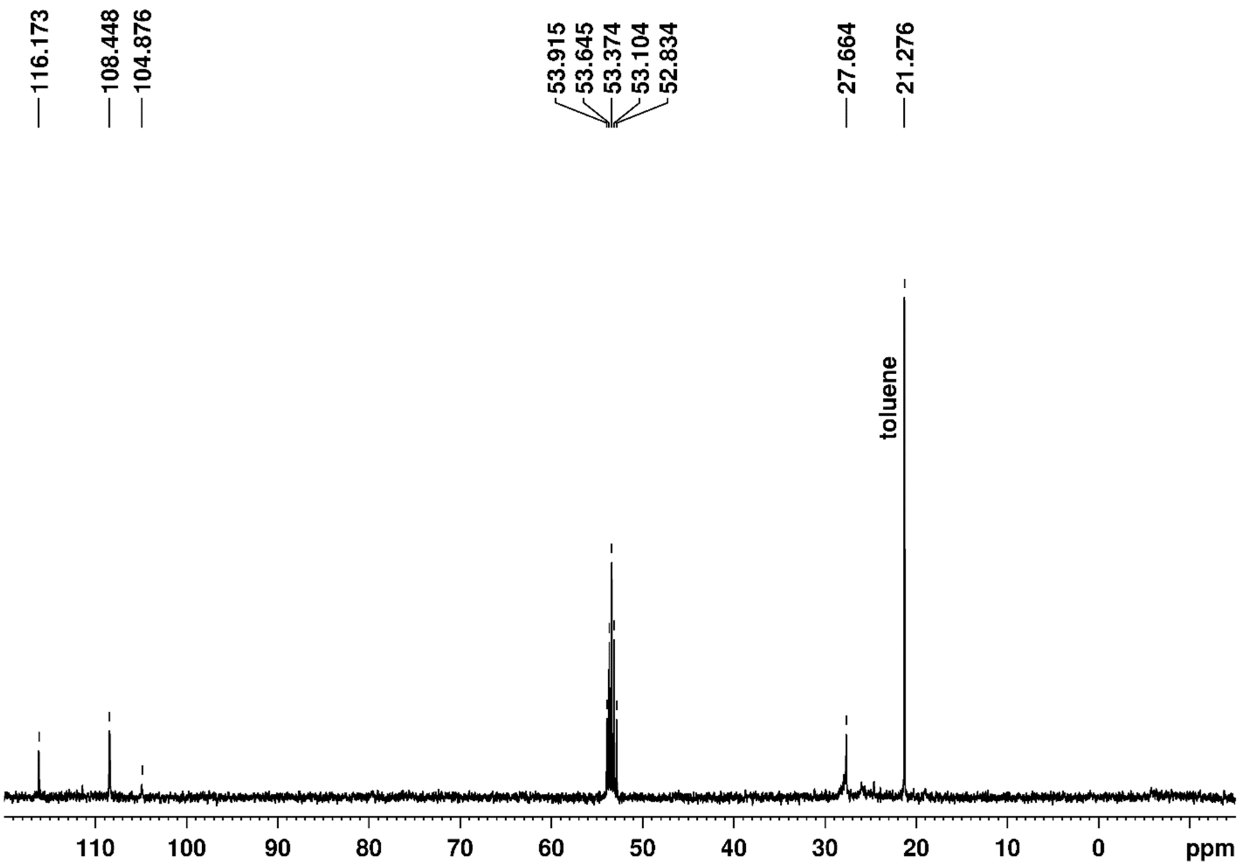

Figure S18. COSY HH NMR of system $[Cp_2ZrH_2]_2$−$ClAlBu^i{}_2$–MMAO-12 (1:2:5) in CD_2Cl_2 (T= 298.7 K).

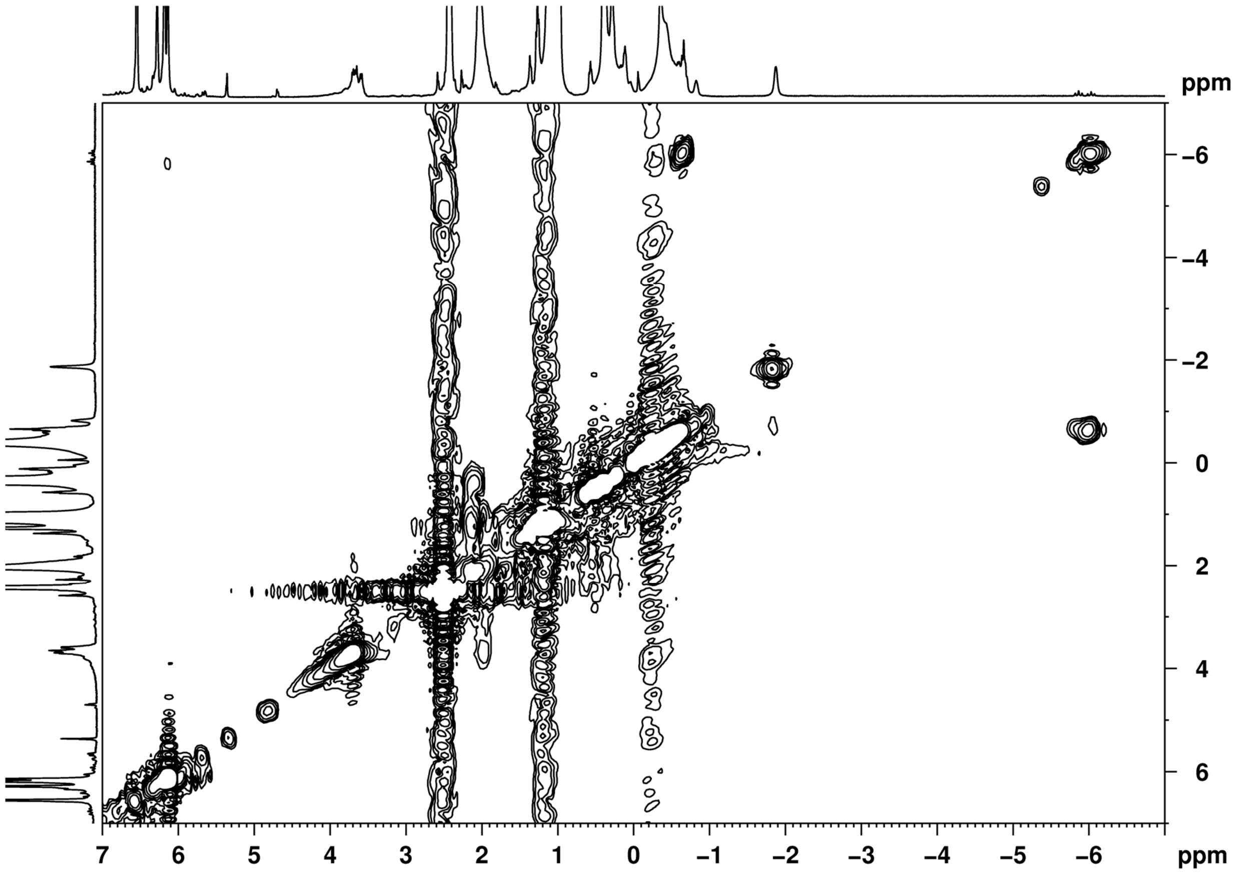

Figure S19. ^{13}C of system $[Cp_2ZrH_2]_2$ – MMAO-12 (1:12) in $CDCl_3$ (T= 296.1 K).

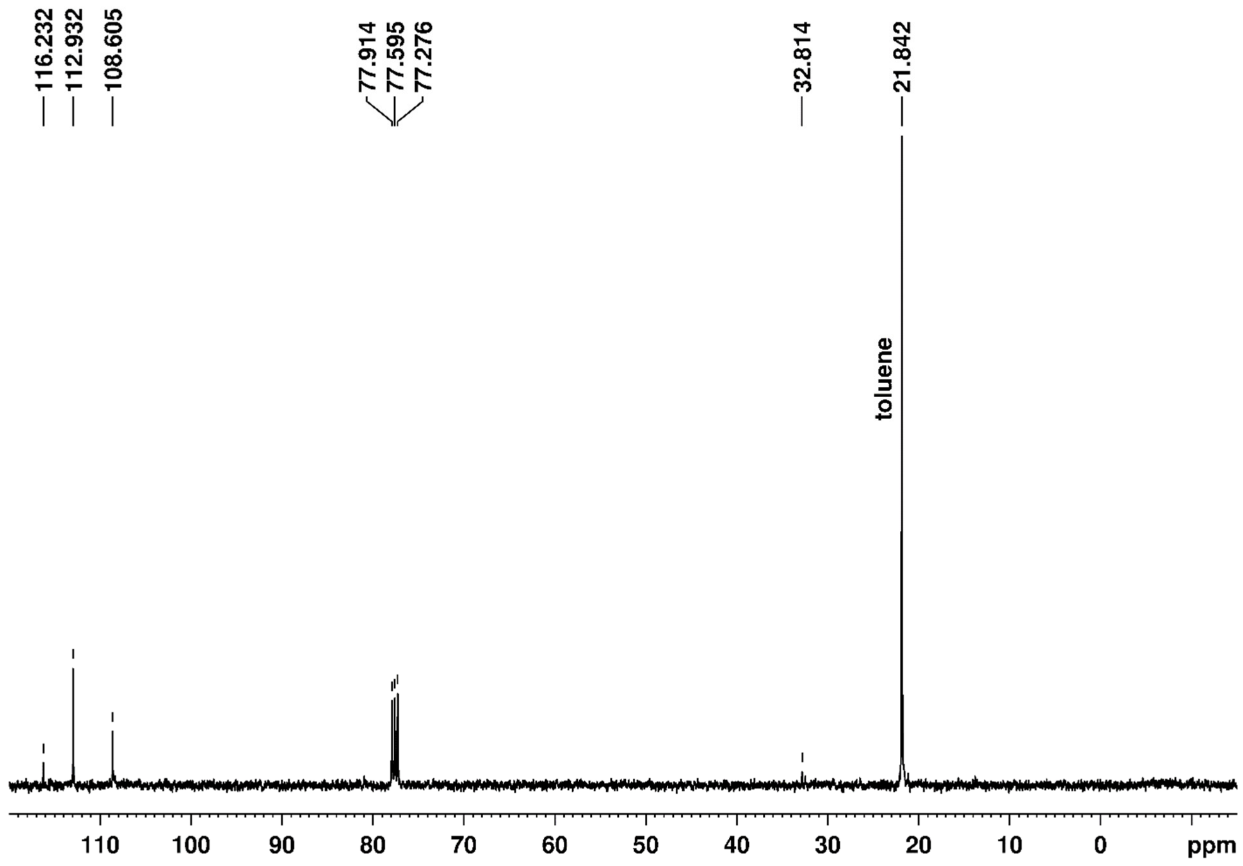

Figure S20. COSY HH NMR of of system $[Cp_2ZrH_2]_2$–MMAO-12 (1:12) in $CDCl_3$ (T= 296 K).

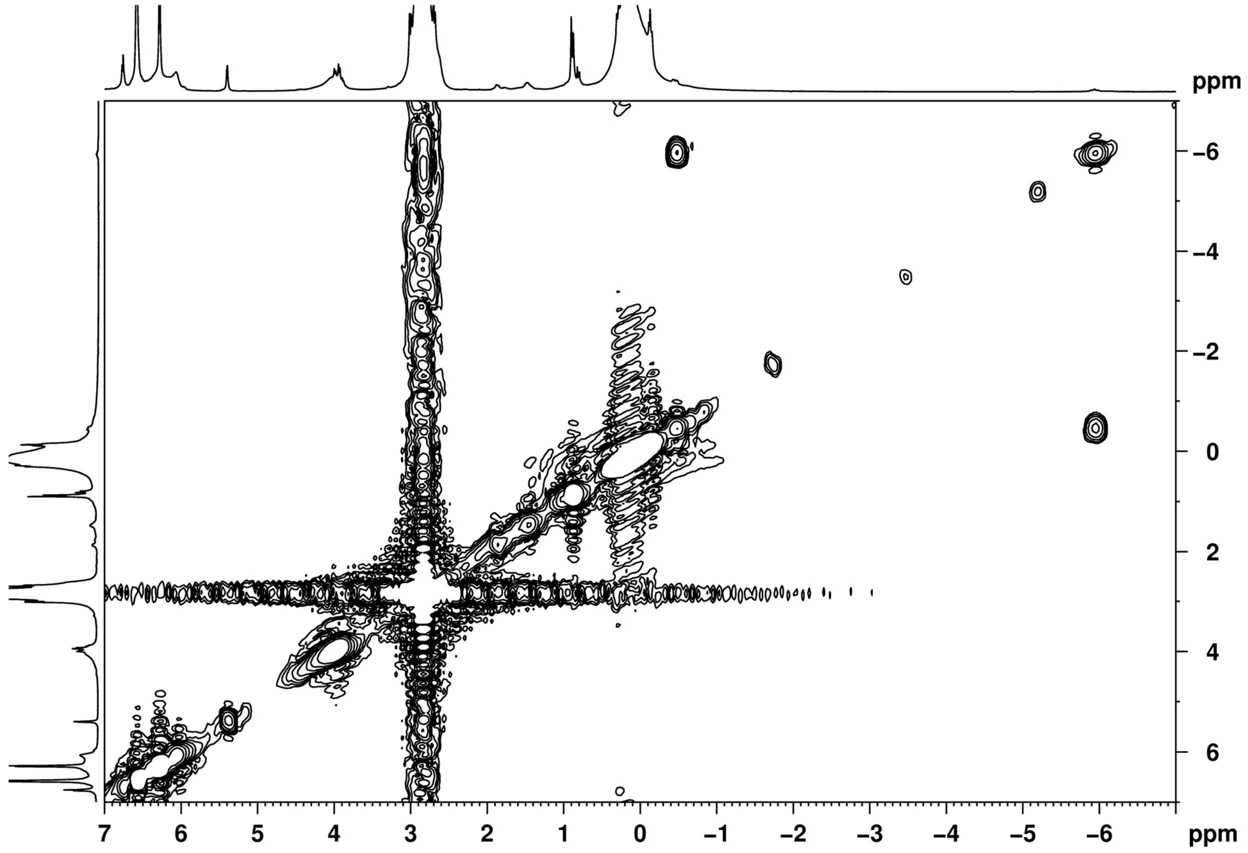

Figure S21. ^{1}H of system $[Cp_2ZrH_2]_2$ – MMAO-12 (1:7) in CD_2Cl_2 (T= 296.3 K).

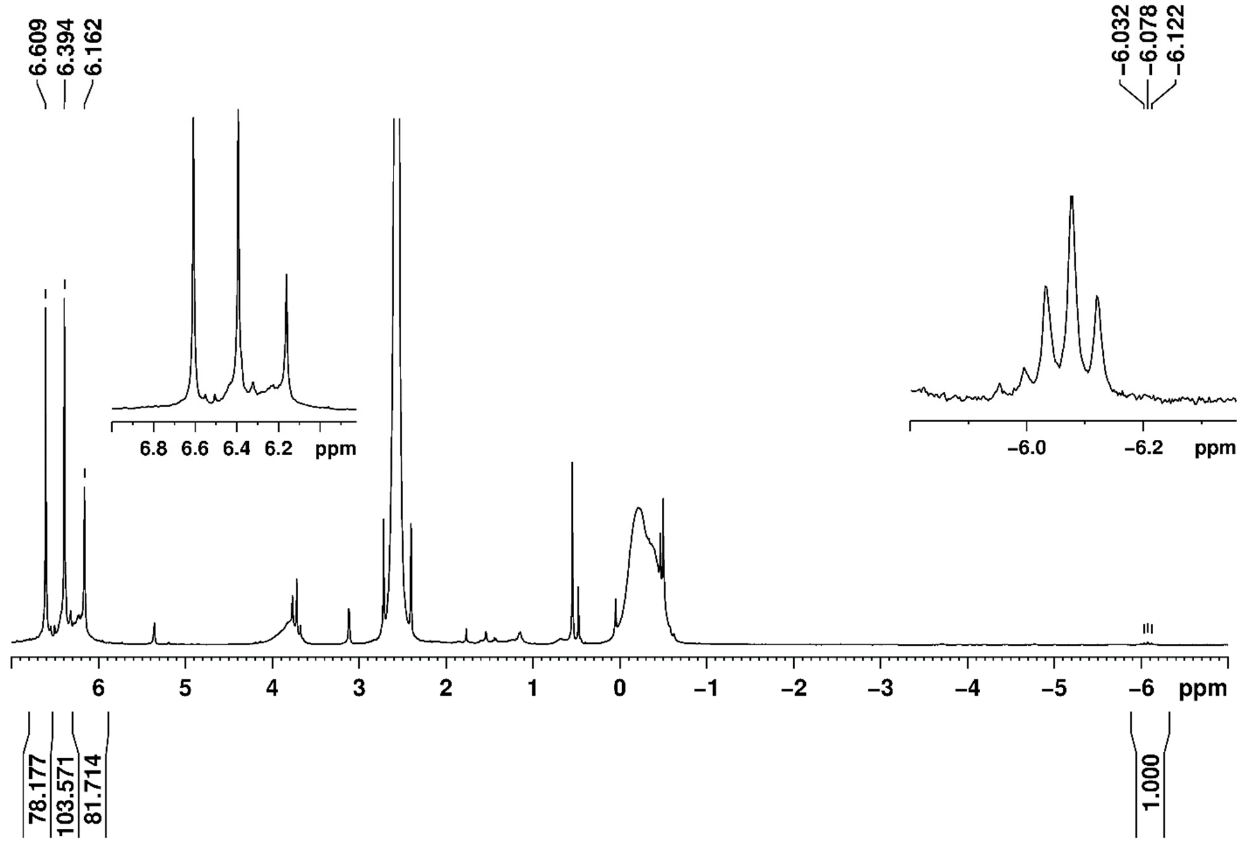

Figure S22. ^{13}C NMR of system $[Cp_2ZrH_2]_2$ – MMAO-12 (1:7) in CD_2Cl_2 (T= 296.6 K).

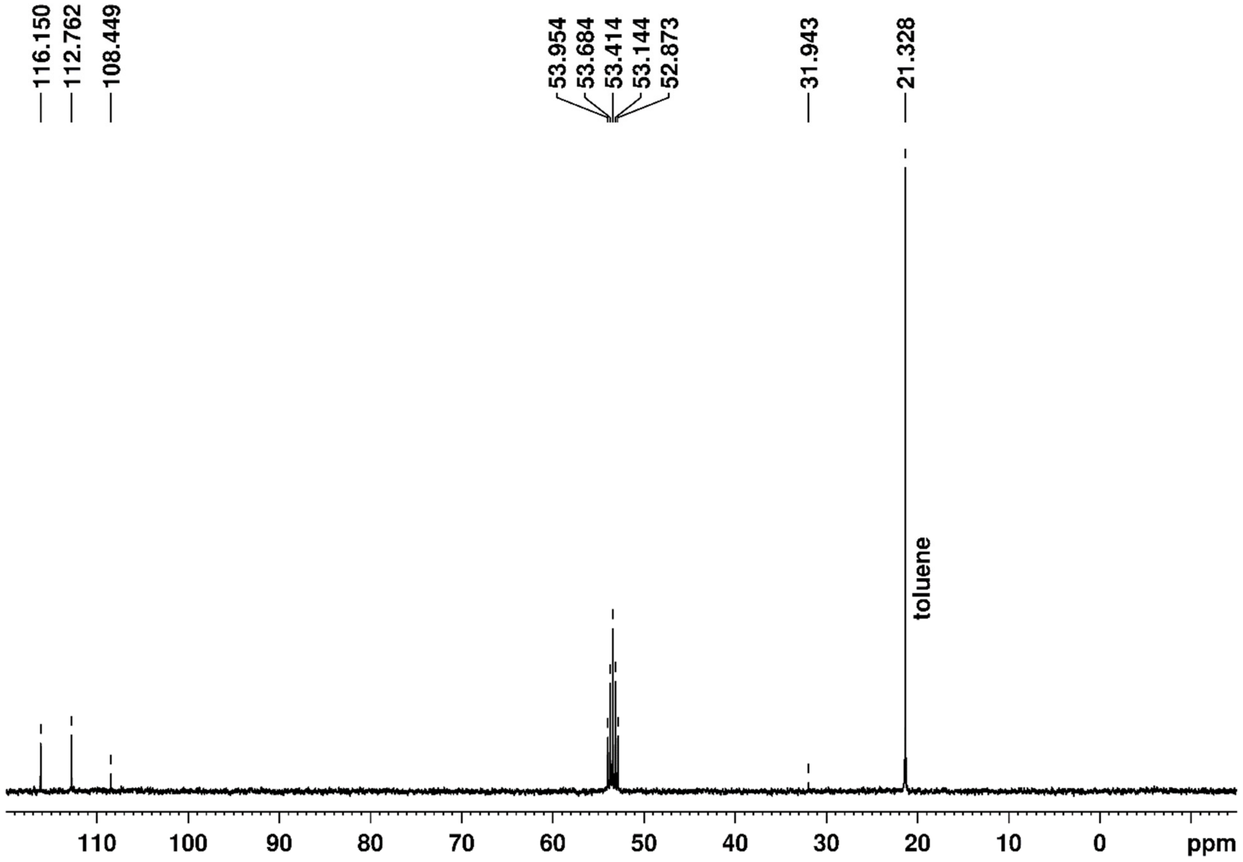

Figure S23. COSY HH of system $[Cp_2ZrH_2]_2$ – MMAO-12 (1:7) in CD_2Cl_2 (T= 296 K)

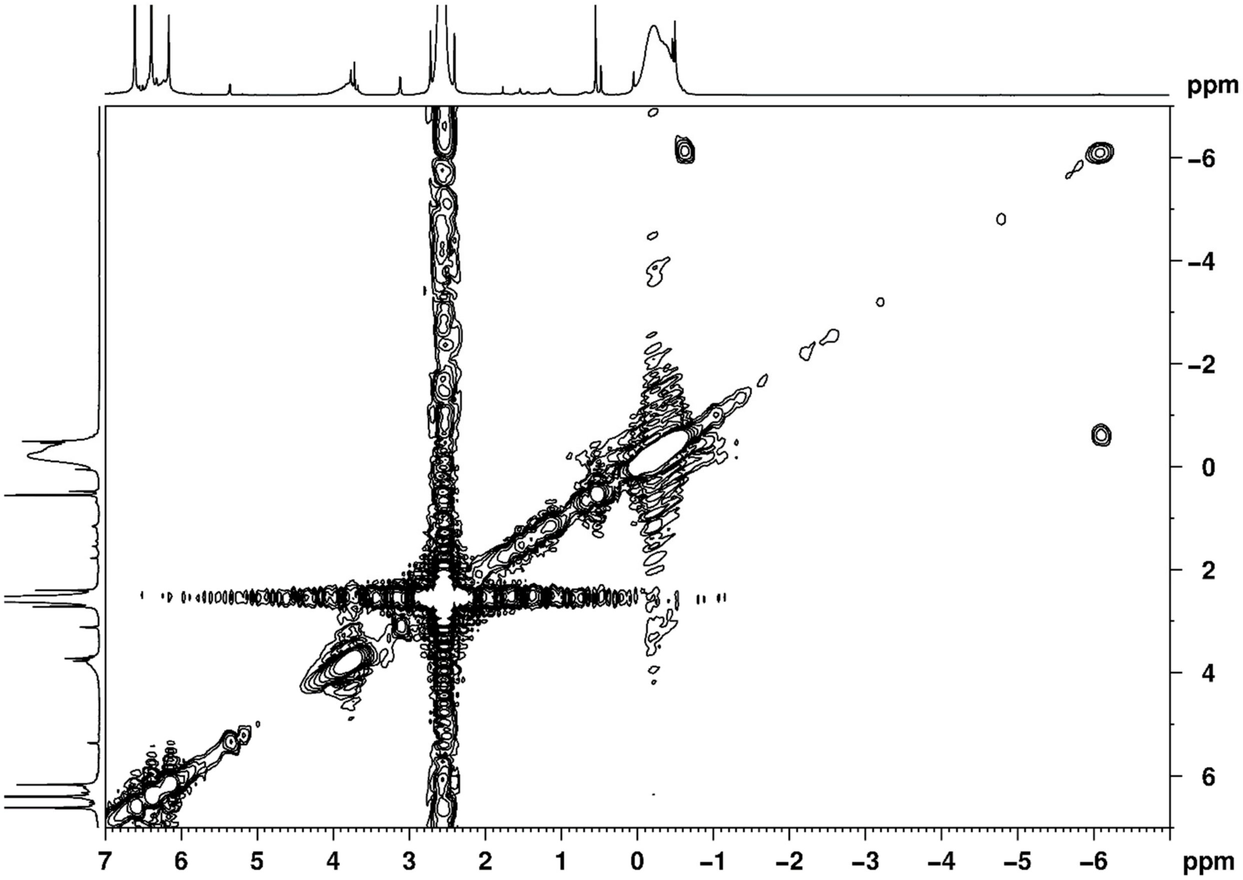

Figure S24. ^{1}H NMR of dimer **5** obtained in the system Cp_2ZrCl_2−$HAlBu^i_2$–MMAO-12, [Zr]:[Al]:[MMAO-12]:[1-alkene]=1:4:8:1 in $CDCl_3$ (T= 298.7 K), 20 min.

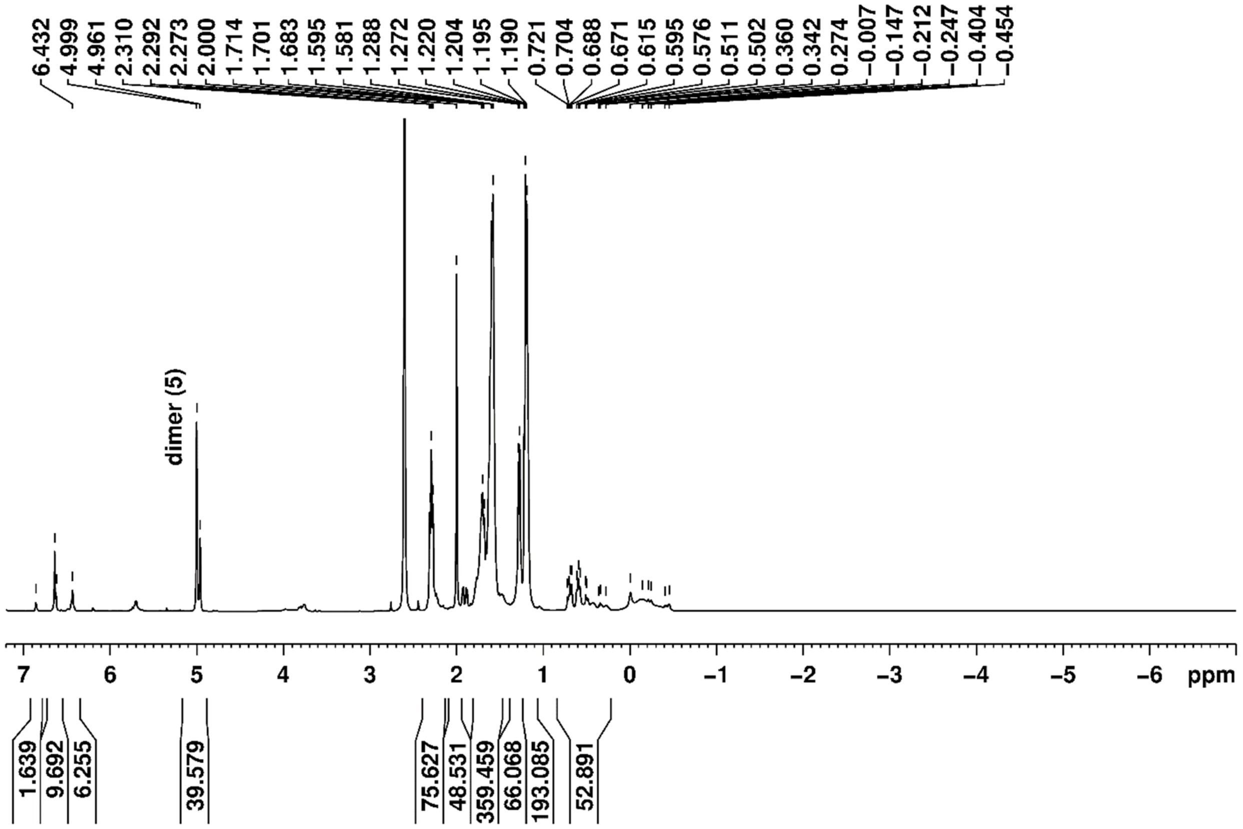

Figure S25. ^{13}C NMR of dimer **5** obtained in the system Cp_2ZrCl_2−$HAlBu^i{}_2$–MMAO-12, [Zr]:[Al]:[MMAO-12]:[1-alkene]=1:4:8:1 in $CDCl_3$ (T= 298.7 K), 20 min.

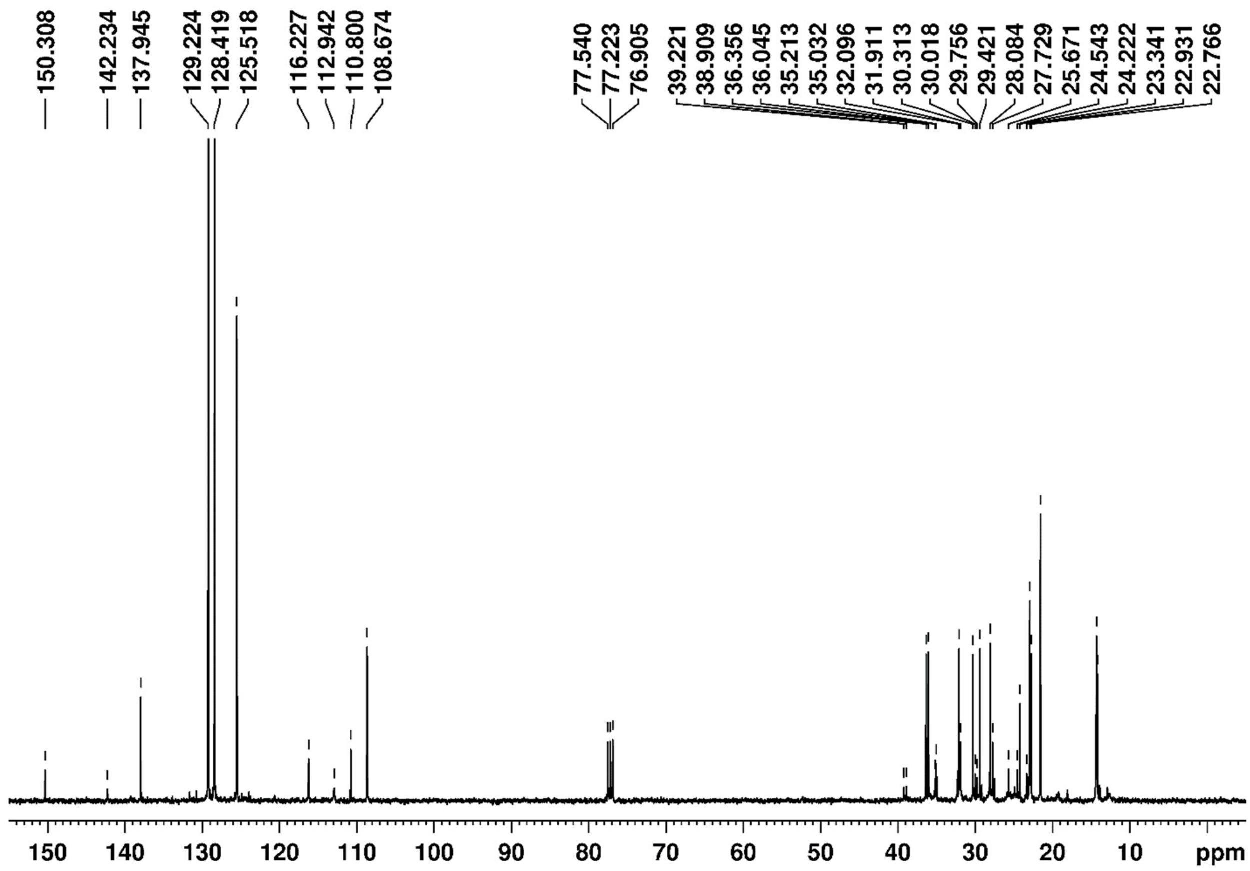

Figure S26. ^{1}H NMR of tetramers **7** obtained in the system Cp_2ZrCl_2–$HAlBu^i_2$–$(Ph_3C)[B(C_6F_5)_4]$, [Zr]:[Al]:[B]:[1-alkene]= 4:16:1:1000, $CHCl_3$, 40°C, 16 h.

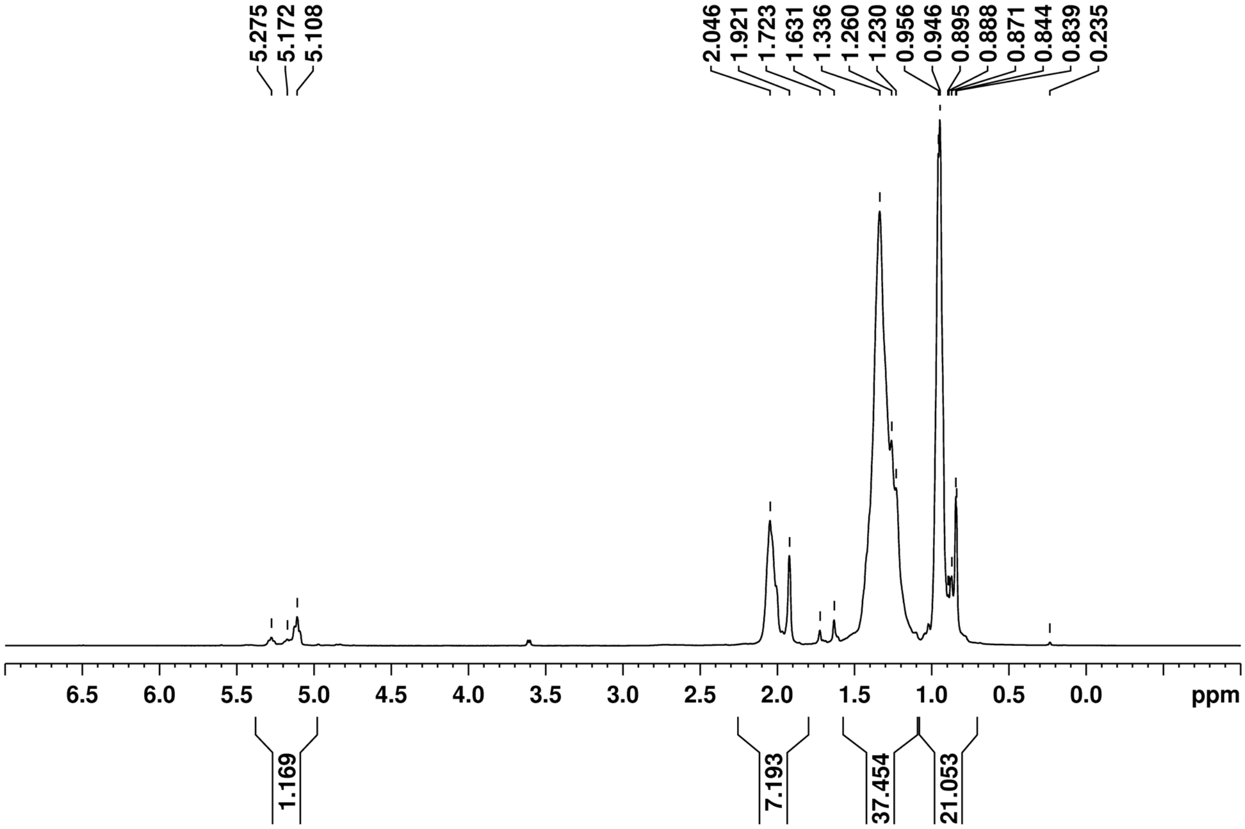

Figure S27. ^{13}C NMR of tetramers **7** obtained in the system Cp_2ZrCl_2–$HAlBu^i_2$–$(Ph_3C)[B(C_6F_5)_4]$, [Zr]:[Al]:[B]:[1-alkene]= 4:16:1:1000, $CHCl_3$, 40°C, 16 h.

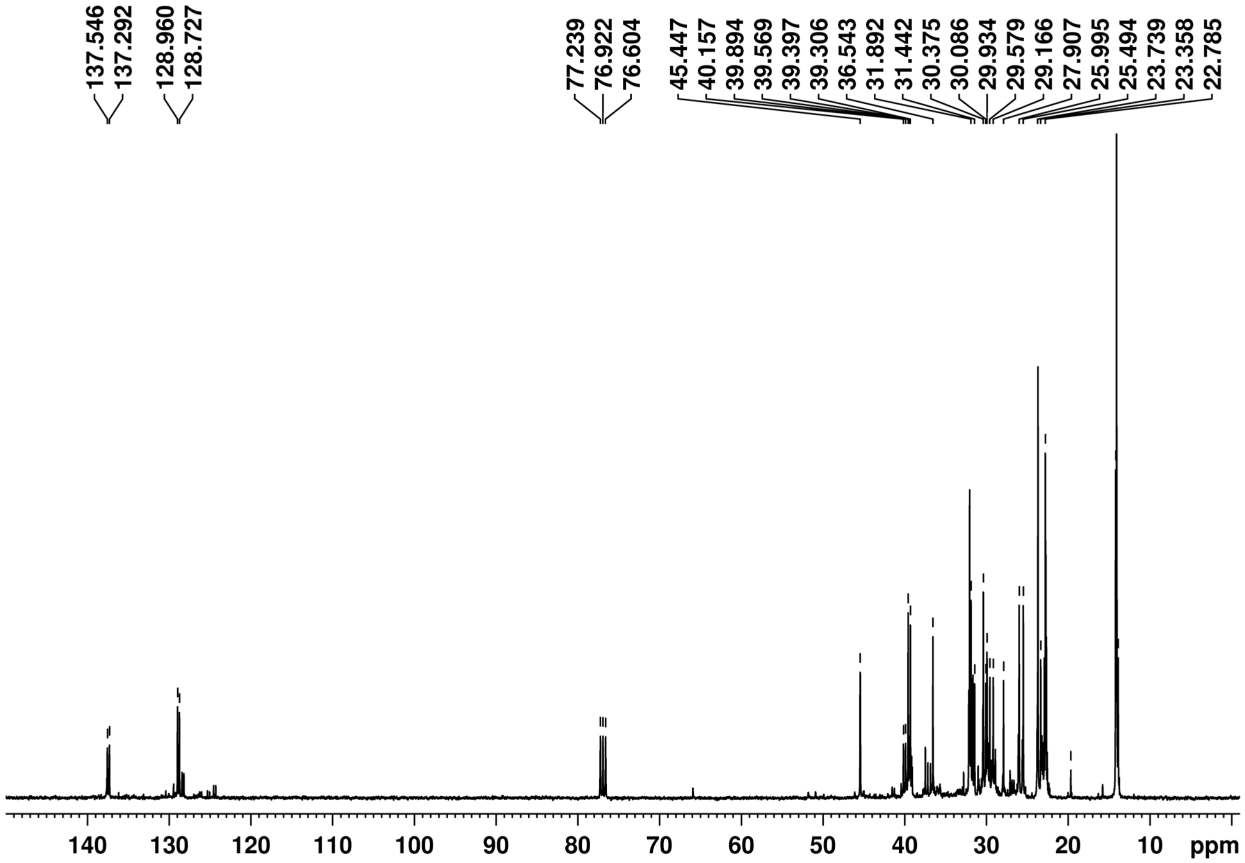

GC-MS analysis of products

Before each series of mass spectral analysis, calibration was performed using 1-hexene – dimer – tetramer mixtures with various mass fractions to determine response factors (RF). Response factors of dimers or tetramers were calculated as RF(dimer or tetramer) = Slope(1-hexene)/Slope(dimer or tetramer), where Slope(1-hexene) was found from the dependence Peak area (1-hexene) – Concentration (1-hexene), and Slope(dimer or tetramer) from the dependence Peak area (dimer or tetramer) – Concentration (dimer or tetramer). 1-Hexene was used as a standard with RF=1. RFs were found for dimer and tetramer as 1.1 and 1.2, correspondingly. Thus, product composition was determined via peak areas multiplied by response factors.

Figure S28. Example of GC-MS analysis of dimers and oligomers obtained in the system $[Cp_2ZrH_2]_2$–$ClAlBu^i{}_2$–MMAO-12, [Zr]:[Al]:[MMAO-12]:[1-alkene]= 1:3:30:1000, CH_2Cl_2, 40°C, 16 h.

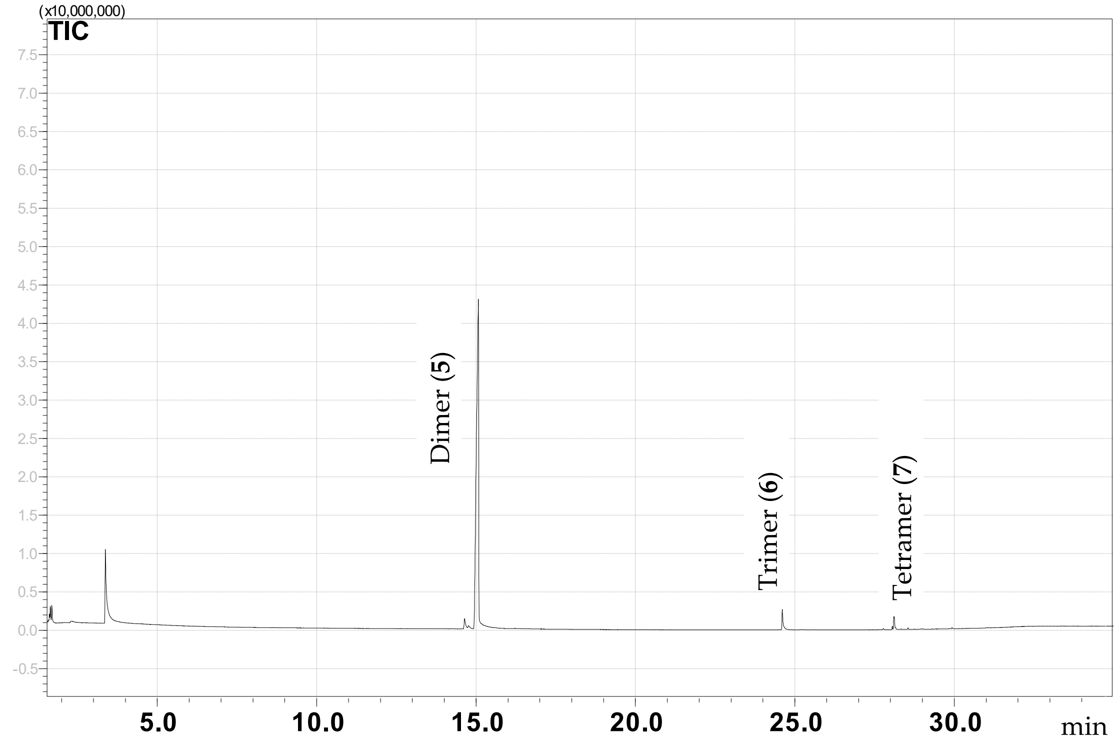

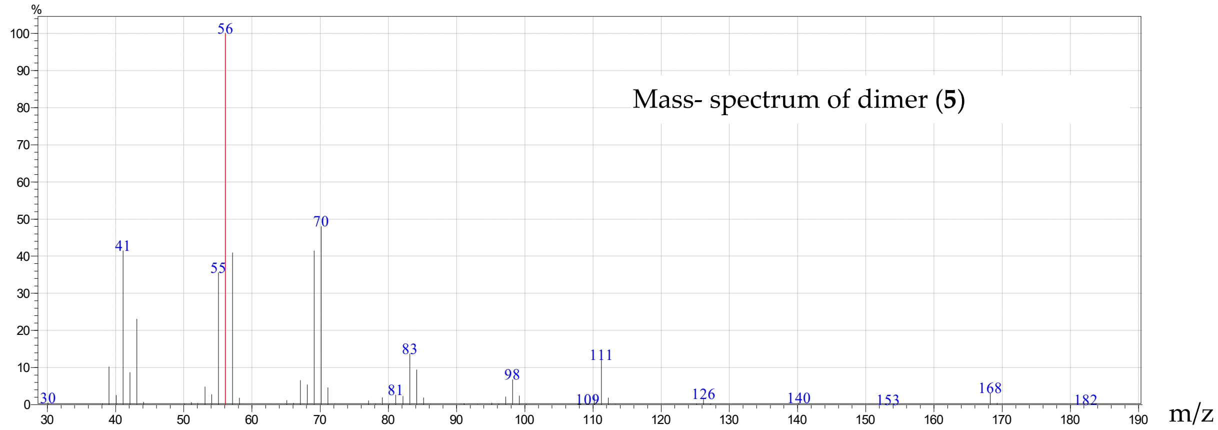

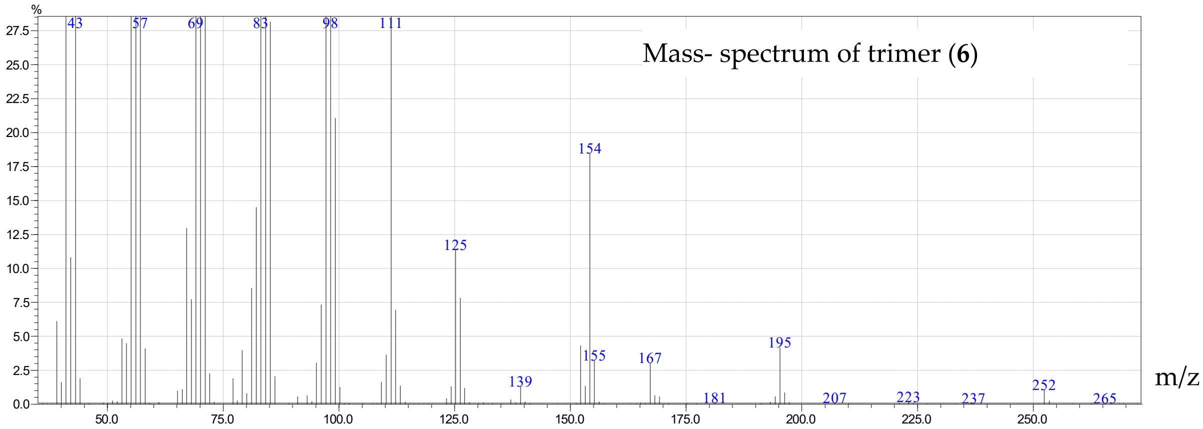

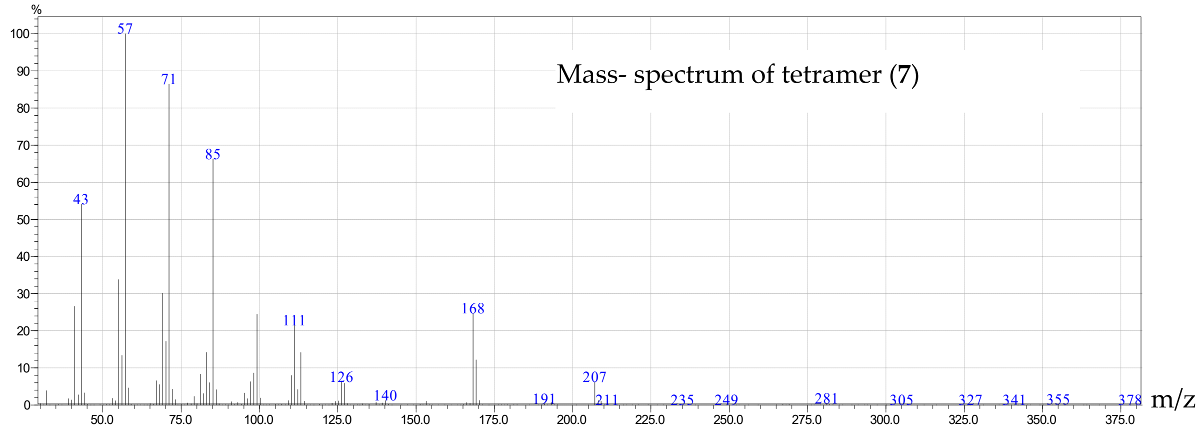

Figure S29. Example of GC-MS analysis of dimers and oligomers obtained in the system Cp_2ZrCl_2–$HAlBu^i_2$–$(Ph_3C)[B(C_6F_5)_4]$, [Zr]:[Al]:[B]:[1-alkene]= 4:16:1:1000, $CHCl_3$, 40°C, 16 h.

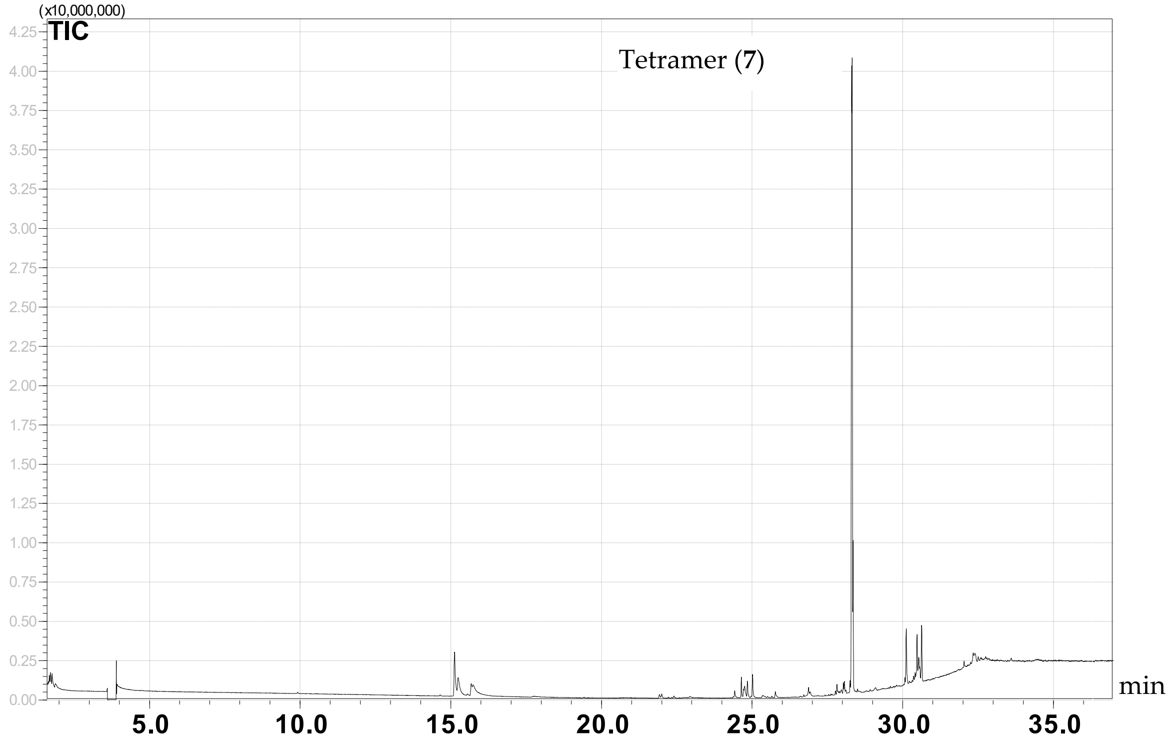

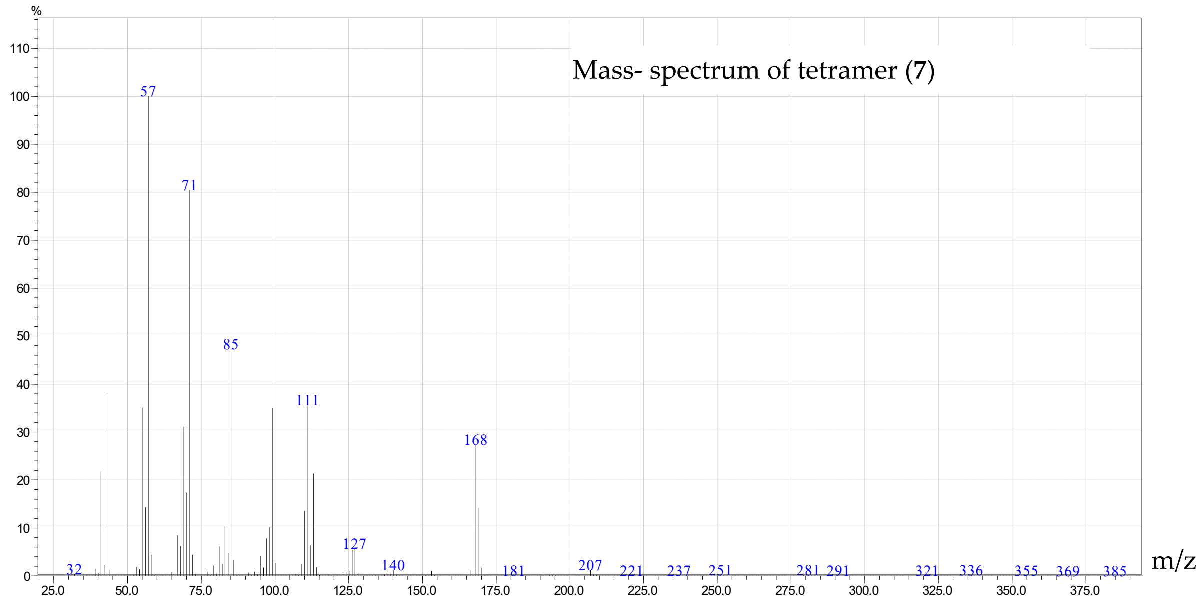
Mass- spectrum of tetramer (7)
%
m/z
57
71
85
111
168
127
140
32
181
207
221
237
251
281
291
321
336
355
369
385

Table S1. The energy at 0K, the ZPVE correction, the enthalpy, the Gibbs free energy in gas, the temperature multiplied by the entropy for complexes **9a-e**, calculated at PBE/3ζ level (298.15 K).

Complex	E, Hartree	ZPVE, Hartree	H, kcal/mol	G, kcal/mol	T*S, cal/mol
**9a**	-9177.483313	0.479046	-5758635.6	-5758702.0	66417.6
**9b**	-9177.470210	0.478892	-5758627.4	-5758692.4	65081.6
**9c**	-9177.480291	0.479422	-5758633.2	-5758698.7	65459.5
**9d**	-9177.461167	0.480010	-5758621.2	-5758685.2	63942.4
**9e**	-10038.215279	0.607638	-6298665.2	-6298747.2	81996.9

Table S2. Relative thermodynamic parameters of isomeric complexes **9a-d**, calculated at PBE0/def2TZVP level (298.15 K).

Complex	ΔE, Hartree	ΔE_{ZPVE}, Hartree	ΔH, kcal/mol	ΔG, kcal/mol	TΔS, cal/mol
9a	0.001420	0.000052	0.0	0.0	2362.8
9b	0.011620	0.010971	6.8	8.4	796.1
9c	0.000000	0.000000	0.0	1.2	1132.1
9d	0.020137	0.020704	12.7	15.1	0.0

Table S3. Relative thermodynamic parameters of isomeric complexes **9a-d**, calculated at PBE0-GD3/def2TZVP level (298.15 K).

Complex	ΔE, Hartree	ΔE_{ZPVE}, Hartree	ΔH, kcal/mol	ΔG, kcal/mol	TΔS, cal/mol
9a	0.001174	0.000050	0.0	0.0	941.6
9b	0.010265	0.009999	6.3	6.5	686.6
9c	0.000000	0.000000	0.1	0.2	898.6
9d	0.020161	0.020487	12.8	13.7	0.0

Table S4. Relative thermodynamic parameters isomeric complexes **9a-d** in $CHCl_3$ solution, calculated at PBE0/def2TZVP level (298.15 K).

Complex	ΔE, Hartree	ΔE_{ZPVE}, Hartree	ΔH, kcal/mol	ΔG, kcal/mol	TΔS, cal/mol
9a	0.000000	0.000000	0.0	0.0	2322.0
9b	0.004409	0.005690	3.6	4.8	1062.3
9c	0.002469	0.002932	2.1	2.3	2049.2
9d	0.021519	0.023216	14.3	16.6	0.0

Table S5. Relative thermodynamic parameters of isomeric complexes **9a-d** in CH_2Cl_2 solution, calculated at PBE0/def2TZVP level (298.15 K).

Complex	ΔE, Hartree	ΔE_{ZPVE}, Hartree	ΔH, kcal/mol	ΔG, kcal/mol	TΔS, cal/mol
9a	0.000000	0.000000	0.0	0.0	2337.8
9b	0.002884	0.004539	2.8	4.2	906.4
9c	0.003194	0.003504	2.5	2.6	2158.6

**9d**	0.022043	0.023679	14.6	17.0	0.0

Atomic coordinates of the complexes **9a-e**

9a

C	3.579096680	-1.182145120	2.772116080
C	2.692045280	-0.086220410	2.960824760
C	3.172921060	1.011578820	2.194632780
C	4.590397310	-0.764442430	1.865318010
C	4.338653910	0.593690410	1.508025440
H	3.513442520	-2.147786050	3.258561900
H	1.811129990	-0.092078430	3.595230600
H	2.712778870	1.990377220	2.127821760
H	5.432051580	-1.365961360	1.539545320
H	4.950478160	1.204947060	0.851304440
Zr	2.462548190	-0.792445320	0.540541630
C	4.139309800	-1.673368520	-1.095318320
C	3.068528890	-2.607475830	-1.100619470
C	1.930701140	-1.951407000	-1.649648020
C	3.662198400	-0.442176090	-1.635319280
C	2.298908610	-0.626303840	-1.991142190
H	5.155272930	-1.873424780	-0.772142620
H	3.116113600	-3.642189800	-0.782739350
H	0.943935190	-2.385835160	-1.769440610
H	4.257567880	0.450215750	-1.802894530
H	1.631910700	0.114671980	-2.416832500
Zr	0.111573650	2.615502270	-0.003216450
H	1.198262360	0.846776720	0.248584470
C	-0.430856440	1.923371560	2.382266270
C	0.181485240	3.203883800	2.437103460
C	-0.701726880	4.133904760	1.812510580
C	-1.670094420	2.057116370	1.707607220
C	-1.847457190	3.425843890	1.359338570
H	-0.023583150	0.991215290	2.756498100
H	1.120841600	3.449678460	2.923135770
H	-0.543793250	5.204137880	1.732225500
H	-2.360003790	1.250456950	1.483885180
H	-2.717353220	3.852942090	0.874816760
C	1.488323490	3.061391500	-2.079418210
C	2.420674990	3.007018930	-1.006043320
C	2.156111090	4.092703050	-0.136521610
C	0.659096420	4.201474080	-1.890339800
C	1.064639800	4.831501140	-0.682535970
H	1.422992680	2.366278930	-2.910712220

H	3.184997340	2.251901720	-0.864975020
H	2.702929040	4.336398020	0.769474130
H	-0.118847430	4.544174980	-2.561736180
H	0.641724300	5.743131690	-0.274502250
H	2.333515050	-2.492526630	1.206988070
H	-1.319248370	3.189002650	-0.991650570
Al	-1.822177370	-1.319379280	-0.393980680
C	-3.516230950	-1.038975000	0.618643650
H	-3.913997300	-2.055846740	0.793019620
H	-3.325191050	-0.640108140	1.628490840
C	-4.587644550	-0.193351680	-0.097418660
H	-4.854604110	-0.621888330	-1.075550840
C	-1.775467240	-2.778490710	-1.751039880
H	-2.758421650	-2.737460640	-2.255608520
H	-1.045028300	-2.564967900	-2.548683080
C	-1.553120880	-4.196539430	-1.189281290
H	-2.319615410	-4.462596020	-0.445331180
H	-1.586663380	-4.968124690	-1.975392870
H	-0.580929420	-4.286745600	-0.681262100
H	-4.236751930	0.831486800	-0.292381350
H	-5.519448880	-0.117079440	0.485913200
Cl	-0.145739730	-1.590898090	1.157978190
Cl	-1.161426150	0.603831900	-1.467065940

9b

C	3.266959420	-1.002395970	3.084015740
C	2.587509850	0.232156330	2.973660540
C	3.338791260	1.066695630	2.090939860
C	4.407401560	-0.960629800	2.242415530
C	4.462717720	0.334401370	1.647112290
H	2.935292050	-1.861456170	3.654725850
H	1.679023630	0.503046940	3.501396750
H	3.103071630	2.087905120	1.818190020
H	5.122251750	-1.765793730	2.102229640
H	5.235494190	0.699934770	0.977702220
Zr	2.435628620	-0.849392570	0.634973800
C	4.298237650	-1.300719400	-1.030216340
C	3.470728160	-2.449762200	-1.061700900
C	2.214337580	-2.070553090	-1.602420490
C	3.550352470	-0.203212160	-1.540685340
C	2.262969190	-0.686639600	-1.903305750
H	5.328292410	-1.268339740	-0.689261140

H	3.730449780	-3.438509220	-0.700764880
H	1.355264680	-2.720363550	-1.724633310
H	3.918097550	0.808612270	-1.671165890
H	1.447993420	-0.103635470	-2.319094840
Zr	0.092466200	2.630544300	-0.157089440
H	1.400333140	0.990403480	0.269793950
C	-0.537606710	1.894459480	2.200754610
C	0.362138450	2.988213050	2.330558530
C	-0.293709810	4.150521280	1.837380160
C	-1.734612630	2.378917870	1.617166270
C	-1.583681370	3.771801810	1.390772500
H	-0.344344560	0.863738240	2.478488930
H	1.354546680	2.957321640	2.766819510
H	0.114916040	5.155978190	1.819981360
H	-2.599869750	1.783585700	1.348781350
H	-2.316845490	4.418928220	0.922574990
C	1.784058440	2.901648270	-2.087019470
C	2.511408360	3.118330030	-0.877232390
C	2.024020600	4.302268290	-0.279339080
C	0.867735450	3.967881460	-2.236862510
C	0.985641570	4.819373670	-1.109517420
H	1.932434350	2.091473910	-2.793406700
H	3.308002730	2.497435100	-0.486640010
H	2.384139790	4.741770430	0.645951390
H	0.146752890	4.077840960	-3.037714860
H	0.403671780	5.718193610	-0.929601710
Al	-1.000600370	-0.751704690	-0.262100140
C	-2.449246580	-1.027123030	1.086687180
H	-2.355833960	-2.087580370	1.383518030
H	-2.289499740	-0.463510310	2.019227010
C	-3.870705580	-0.772596950	0.545665490
H	-4.078140210	-1.380649170	-0.347717770
C	-1.257218310	-1.713885360	-1.994721740
H	-2.130258870	-1.221364440	-2.460044320
H	-0.434624740	-1.551509520	-2.708704120
C	-1.543779770	-3.221639010	-1.844013400
H	-2.415533400	-3.404452580	-1.197900910
H	-1.753259530	-3.705878470	-2.811657020
H	-0.701221540	-3.761698630	-1.384450760
H	-4.018965800	0.276312900	0.244693700
H	-4.649644010	-1.012860610	1.287296930
H	0.453452120	-1.011227640	0.585168260

H	-0.614857110	0.849874560	-0.692421670
Cl	-1.960871720	2.959401590	-1.623916490
Cl	1.774391860	-3.154065270	1.486592000

9c

C	1.955884260	-2.435907860	1.283024320
C	1.939686710	-1.309157740	2.132779030
C	3.213285760	-0.679038880	2.045135630
C	3.256805420	-2.542286060	0.706967450
C	4.035219070	-1.473874390	1.195919030
H	1.126879300	-3.114283010	1.109592410
H	1.101789480	-0.981130650	2.734467350
H	3.536409250	0.203175410	2.589958260
H	3.579422690	-3.316170710	0.017158420
H	5.073648400	-1.271539000	0.957708200
Zr	2.138212940	-0.341510100	-0.273776410
C	4.280776990	-0.990240250	-1.827475390
C	3.117699910	-1.018482900	-2.630722400
C	2.608843830	0.310269570	-2.723541500
C	4.456360530	0.331770410	-1.358009590
C	3.433724720	1.142961420	-1.934508350
H	4.892461680	-1.847609010	-1.568797140
H	2.699561200	-1.891740720	-3.122864080
H	1.732298090	0.609911930	-3.288166980
H	5.251470460	0.687658160	-0.709137260
H	3.328347570	2.212132740	-1.795964130
Zr	0.184707130	2.116141470	0.461459830
H	2.091634580	1.492611190	0.544371710
C	0.694260520	1.828025700	2.969235190
C	0.590243470	3.243172800	2.820668270
C	-0.766269520	3.557021650	2.571800420
C	-0.590345360	1.273123930	2.771074170
C	-1.488797590	2.344656600	2.493516010
H	1.601281240	1.286959560	3.210855580
H	1.408465150	3.949395760	2.928714830
H	-1.173056200	4.548784570	2.406468860
H	-0.857718420	0.222766630	2.812166650
H	-2.550318910	2.242804600	2.288130860
C	0.743191650	3.311708990	-1.783534890
C	1.470975080	3.984984480	-0.760968430
C	0.536968520	4.709560180	0.033979610
C	-0.629453610	3.558455990	-1.569235190

C	-0.757941730	4.429088550	-0.445729000
H	1.160700310	2.708232360	-2.579149760
H	2.550885070	4.016107150	-0.649276940
H	0.788152280	5.348202870	0.873547200
H	-1.446180560	3.167023220	-2.166869980
H	-1.693483380	4.807131350	-0.045051330
Al	-1.932656930	-1.427888800	-0.570454440
H	0.535590600	0.170037730	0.757732710
C	-3.201583660	-1.926850960	-2.010666620
H	-4.193140120	-1.520013100	-1.744679710
H	-2.908638240	-1.405687870	-2.937190650
C	-3.314042350	-3.442368630	-2.270364540
H	-3.638991650	-3.987298270	-1.370685890
C	-2.033332540	-2.203806950	1.253121820
H	-1.913731760	-3.297047900	1.146222330
H	-1.174853960	-1.875861850	1.860828510
C	-3.352055150	-1.901877570	1.992433940
H	-4.225262240	-2.259636610	1.425889250
H	-3.394919510	-2.377858680	2.985149100
H	-3.495053020	-0.821052660	2.144076100
H	-2.348913810	-3.875186010	-2.574845020
H	-4.037972040	-3.676054750	-3.067322290
H	0.863815650	1.001652410	-1.005008800
Cl	0.176692580	-1.792489120	-1.440712820
Cl	-2.039213920	0.879305830	-0.466328200

9d

C	2.074018750	-2.321192560	1.452944550
C	2.108529230	-1.094481620	2.166423580
C	3.411330360	-0.552685020	2.049592900
C	3.368721440	-2.547207280	0.918468400
C	4.193596820	-1.450912360	1.278909730
H	1.220334600	-2.983965860	1.367179210
H	1.282944760	-0.665646600	2.714415560
H	3.764436180	0.383505450	2.468795230
H	3.665541260	-3.415031810	0.340107040
H	5.240586140	-1.317112710	1.028166870
Zr	2.431852200	-0.515144780	-0.353428080
C	4.414490910	-1.659887300	-1.689979290
C	3.200605070	-2.113719040	-2.262301770
C	2.584873140	-1.004221990	-2.911624820
C	4.510488820	-0.264709340	-1.904584970

C	3.389213130	0.132976250	-2.689940690
H	5.131337000	-2.268843330	-1.151715080
H	2.827464090	-3.133873470	-2.260310910
H	1.642293700	-1.028539080	-3.446677090
H	5.317802460	0.382705940	-1.572822230
H	3.170693080	1.143304250	-3.015539430
Zr	0.155983490	2.450917210	0.500237510
H	0.717868140	0.334256410	0.572670530
C	0.928703400	1.978249010	2.918470390
C	0.701216530	3.383823300	2.847036550
C	-0.693699120	3.586585220	2.723791500
C	-0.313629440	1.325965380	2.784119790
C	-1.319533850	2.322916460	2.626443040
H	1.897596760	1.510041710	3.033731720
H	1.462108190	4.155277630	2.922923820
H	-1.196903310	4.544801230	2.673440520
H	-0.502897760	0.259567680	2.772500750
H	-2.381628400	2.135374580	2.502719150
C	-0.004823220	3.881346900	-1.773759690
C	0.693022500	4.667141800	-0.826318230
C	-0.209238590	4.997816380	0.218931730
C	-1.316762010	3.662990760	-1.289559010
C	-1.449694680	4.367470760	-0.058474880
H	0.410984210	3.474765730	-2.685753120
H	1.729377750	4.984230070	-0.898938120
H	0.020650270	5.633233070	1.066682210
H	-2.090148660	3.077962910	-1.777234820
H	-2.355610050	4.429852840	0.535862060
H	0.793083860	-1.494407250	-0.650270600
H	-1.286006800	1.206750360	0.165549290
Al	-0.787973780	-0.571485790	-0.216872810
C	-2.029383550	-0.964176970	-1.755242800
H	-1.692026850	-0.470887340	-2.677055650
H	-1.966049960	-2.050252010	-1.947272890
C	-3.492018520	-0.581717540	-1.460717750
H	-3.604682890	0.506067250	-1.329563410
C	-1.423675400	-1.640543050	1.386079370
H	-0.697555010	-1.687631430	2.214369020
H	-2.308298150	-1.126586390	1.805380160
C	-1.825559160	-3.081133540	1.008403070
H	-0.981504880	-3.644405450	0.577218690
H	-2.192944640	-3.659652000	1.872073360

H	-2.622247820	-3.094280710	0.250197300
H	-3.865427380	-1.050347090	-0.536353310
H	-4.176714880	-0.882023760	-2.271443780
Cl	0.583984120	1.030112750	-1.885778890
Cl	2.810437230	2.096560320	0.236026810

9e

C	1.381648820000	-2.899962100000	0.570998160000
C	1.068028380000	-2.035530300000	1.658575810000
C	2.291159220000	-1.554618830000	2.194710620000
C	2.791769350000	-2.931971520000	0.423728140000
C	3.357673060000	-2.089301300000	1.427075470000
H	0.656961620000	-3.448217950000	-0.020966690000
H	0.060887150000	-1.824796550000	2.007148700000
H	2.405393720000	-0.868230870000	3.026992470000
H	3.342426650000	-3.512159750000	-0.310005240000
H	4.414962430000	-1.919728920000	1.605544630000
Zr	2.136188100000	-0.589917420000	-0.153360780000
C	3.601066560000	-1.117101770000	-2.151277160000
C	2.767782540000	-0.039760980000	-2.571697120000
C	3.107067390000	1.096580380000	-1.804664670000
C	4.492759880000	-0.620264370000	-1.161991230000
C	4.178127990000	0.737987440000	-0.929526860000
H	3.582092780000	-2.122708200000	-2.557869930000
H	1.993629140000	-0.083956890000	-3.333073380000
H	2.653099230000	2.078007310000	-1.885910960000
H	5.272471210000	-1.187234530000	-0.662885710000
H	4.669211030000	1.395956090000	-0.220008500000
Zr	-0.435929980000	1.829457920000	1.191220340000
Cl	2.205613320000	1.601284540000	1.345931560000
C	-0.162027830000	1.046478110000	3.604520540000
C	0.190211760000	2.431175660000	3.595781750000
C	-0.976375010000	3.174483820000	3.305067320000
C	-1.542128580000	0.942923960000	3.322100270000
C	-2.044071170000	2.258375540000	3.100662050000
H	0.507153340000	0.220736450000	3.819731890000
H	1.177190650000	2.839186470000	3.787917590000
H	-1.042075300000	4.256134310000	3.240079700000
H	-2.110754870000	0.018572290000	3.266446320000
H	-3.073570490000	2.507768800000	2.866351960000
C	-0.431697040000	2.500410720000	-1.271934760000
C	0.344613620000	3.471434430000	-0.586858230000

C	-0.501788640000	4.167011200000	0.314489420000
C	-1.774910810000	2.617065950000	-0.812936880000
C	-1.820431830000	3.641253240000	0.166769440000
H	-0.099334190000	1.799345460000	-2.032329510000
H	1.410098830000	3.642003140000	-0.698653990000
H	-0.202437530000	4.983605640000	0.964114110000
H	-2.612278510000	2.030575330000	-1.174881920000
H	-2.706713600000	3.978423150000	0.694984400000
H	0.803951950000	-1.308699560000	-1.366473470000
H	-1.837545470000	0.567770820000	0.736753430000
Al	-3.434948290000	-0.331341040000	0.680040410000
H	0.255808370000	0.189214930000	0.156671120000
C	-3.816939520000	-0.557726470000	-1.261983300000
H	-2.892105430000	-0.470674170000	-1.852939060000
H	-4.131858860000	-1.609060060000	-1.380330040000
Al	-0.000154510000	-2.161372900000	-2.775344690000
C	-4.797333700000	0.606577880000	1.805908070000
H	-4.445509370000	0.762415390000	2.837576780000
H	-5.002657920000	1.610175590000	1.391648890000
C	-1.439152980000	-3.263341650000	-1.947697000000
H	-1.704961330000	-2.872753620000	-0.953366560000
H	-2.335470740000	-3.094687030000	-2.569357610000
C	1.369903340000	-3.087792430000	-3.901994460000
H	1.878147850000	-3.875701830000	-3.317649890000
H	2.156162230000	-2.396074610000	-4.242562910000
Cl	-0.691367960000	-0.325281960000	-3.806228420000
Cl	-2.725647630000	-2.154333240000	1.721001280000
C	0.703455350000	-3.717924540000	-5.145314320000
H	1.430319870000	-4.243032840000	-5.786052310000
H	-0.071572280000	-4.449986830000	-4.872696460000
H	0.216372800000	-2.952353480000	-5.767601920000
C	-1.164075520000	-4.777244280000	-1.858865740000
H	-2.001596220000	-5.317710730000	-1.389311800000
H	-1.004148700000	-5.226453780000	-2.850198150000
H	-0.266796020000	-5.013839370000	-1.264175910000
C	-4.903111010000	0.363970510000	-1.850247890000
H	-5.065967340000	0.170795570000	-2.922600220000
H	-5.872786200000	0.232165850000	-1.347487560000
H	-4.650675940000	1.432838330000	-1.759041500000
C	-6.108778410000	-0.208397860000	1.854344460000
H	-6.531473730000	-0.371337790000	0.851438220000
H	-5.944108700000	-1.201710190000	2.297858090000

H	-6.887151400000	0.290601530000	2.454109550000
